# Differential expression profiles and pathways of genes in sugarcane leaf at elongation stage in response to drought stress

**DOI:** 10.1038/srep25698

**Published:** 2016-05-12

**Authors:** Changning Li, Qian Nong, Manoj Kumar Solanki, Qiang Liang, Jinlan Xie, Xiaoyan Liu, Yijie Li, Weizan Wang, Litao Yang, Yangrui Li

**Affiliations:** 1 Key Laboratory of Sugarcane Biotechnology and Genetic Improvement (Guangxi), Ministry of Agriculture, Guangxi Key Laboratory of Sugarcane Genetic Improvement, Sugarcane Research Center of Chinese Academy of Agricultural Sciences, Sugarcane Research Institute of Guangxi Academy of Agricultural Sciences, Nanning, Guangxi 530007, China; 2 Microbiology Research Institute of Guangxi Academy of Agricultural Sciences, Nanning, Guangxi 530007, China; 3 College of Agriculture, State Key Laboratory of Conservation and Utilization of Subtropical Agro-bioresources, Guangxi University, Nanning, Guangxi 530004, China

## Abstract

Water stress causes considerable yield losses in sugarcane. To investigate differentially expressed genes under water stress, a pot experiment was performed with the sugarcane variety GT21 at three water-deficit levels (mild, moderate, and severe) during the elongation stage and gene expression was analyzed using microarray technology. Physiological parameters of sugarcane showed significant alterations in response to drought stress. Based on the expression profile of 15,593 sugarcane genes, 1,501 (9.6%) genes were differentially expressed under different water-level treatments; 821 genes were upregulated and 680 genes were downregulated. A gene similarity analysis showed that approximately 62.6% of the differentially expressed genes shared homology with functional proteins. In a Gene Ontology (GO) analysis, 901 differentially expressed genes were assigned to 36 GO categories. Moreover, 325 differentially expressed genes were classified into 101 pathway categories involved in various processes, such as the biosynthesis of secondary metabolites, ribosomes, carbon metabolism, etc. In addition, some unannotated genes were detected; these may provide a basis for studies of water-deficit tolerance. The reliability of the observed expression patterns was confirmed by RT-PCR. The results of this study may help identify useful genes for improving drought tolerance in sugarcane.

Abiotic stress is a major constraint for crop production worldwide, reducing total yields by more than 50%^1^. Sugarcane (*Saccharum* spp.) is a major sugar crop grown in tropical and sub-tropical areas throughout the world; it is vulnerable to adverse environmental conditions, especially abiotic stresses, such as water stress and low temperatures^2^. It is important to breed new sugarcane varieties that are able to use water efficiently or tolerate/resist drought. In response to extreme environments, plants have developed numerous adaptive systems, including metabolic, cellular, and physiological processes, to promote acclimatization and survival. Morphological and physiological modifications are the first weapons that plants use to protect against drought stress^3^. Moreover, under desiccation conditions, plants may increase the synthesis of peroxidases^4^, heat shock proteins^5^, water transport proteins^6^, and loci involved in proteolysis^7^. Moreover, molecular responses, like the expression of multiple genes (pathways associated with stress regulation), signal transduction, proteins, compounds synthesis, and regulatory loci, also play important roles in drought stress^8^.

Plant responses usually depend on the drought intensity, duration, and rate of progression. Abscisic acid (ABA) is an important plant hormone that plays a major role in cell signaling and gene regulation^9^. Cutler *et al*. reported that the endogenous ABA concentration increases under water-deficit conditions and regulates plant stomata functions to protect against instant desiccation^9^. Gene expression studies of exogenous ABA treatment under water-deficit conditions have identified groups of genes that share similar molecular mechanisms of abiotic stress responses^10^.

In the past few decades, breeders have attempted to understand the tolerance process in order to develop more resistant cultivars. In crops like sugarcane, improvements through a conventional breeding approach are difficult owing to its genetic complexity and polyploid nature with significant levels of chromosomal mosaicism^11^. A modern sugarcane cultivar is an inter-specific hybrid derived from crosses between true sugarcane, *Saccharum officinarum*, and *S. spontaneum*/*barberi*/*sinense*. Moreover, drought stress tolerance in plants is a complex quantitative trait regulated by many genes^12^. Conventional breeding strategies for crop improvement are limited by the complexity of stress-tolerance traits, a lack of efficient selection techniques, and low genetic variance and fertility^13^. Differentially expressed gene profiling is an alternative way to identify genes and pathways related to stress responses using modern molecular techniques, such as microarrays. This technique may allow plant breeders to manipulate only a characteristic of interest^14^. The microarray technique has been used to identify regulatory pathways involved in salt stress responses in *Medicago truncatula*^15^ and to evaluate the effect of water stress on barley^16^. This method has also been utilized to study the photoperiod influence under water stress and to analyze the metabolic pathways regulated by jasmonates in *Arabidopsis thaliana*^17^. In sugarcane, this technology was used to study low potassium stress^18^, photosynthesis^19^, and water stress responses^20^. It is important to isolate genes related to water deficit responses and to study the associated pathways in order to develop drought-tolerant sugarcane varieties. In the present study, our goal was to identify differentially expressed genes at the elongation stage, a key stage determining yield in sugarcane, under water-deficit conditions using a microarray approach and to reveal the associated pathways; the results were validated by examining gene expression using qRT-PCR.

## Results

### Changes in physiological parameters under water stress

**Figure 1:**
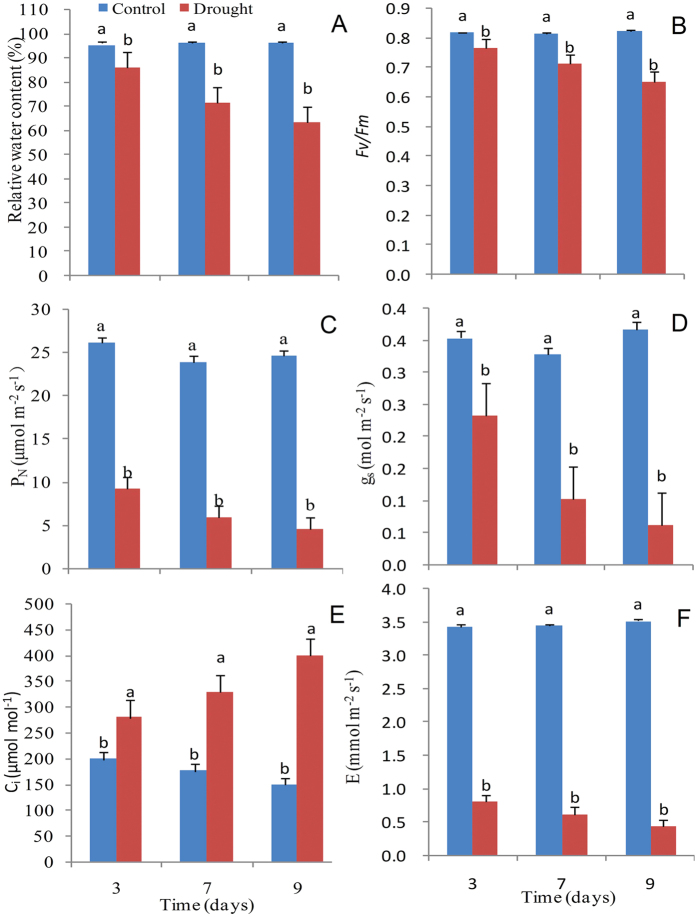
Effects of drought stress on different physiological parameters in leaves of sugarcane. (**A**) Leaf relative water content (RWC), (**B**) Maximum photochemical efficiency (*Fv/Fm*), (**C**) Net photosynthetic rate (P_n_), (**D**) Stomatal conductance (g_s_), (**E**) Intercellular CO_2_ concentration (C_i_), (**F**) Transpiration rate. Drought-stressed plants were collected on the 3^rd^, 7^th^ and 9^th^ days, which corresponded to mild, moderate, and severe water stress conditions, respectively. Bars with different superscript letters indicate significant differences at the P < 0.05 probability at the same time point.

**Figure 2:**
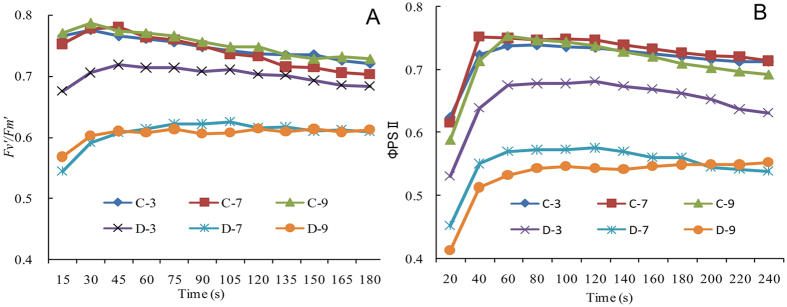
Effects of drought stress on *Fv*′/*Fm*′ (**A**) and ΦPSII (**B**) in leaves of sugarcane. C-3, C-7, and C-9 represent samples obtained on days 3,7 and 9 for the control, and D-3, D-7, and D-9 represent samples obtained on days 3,7 and 9 for drought-stressed plants. Drought-stressed plants were collected on the 3^rd^, 7^th^ and 9^th^ days, which corresponded to mild, moderate, and severe water stress conditions, respectively.

Physiological parameters of sugarcane plants were determined to verify the effects of water stress. The leaf relative water content decreased by 25% during mild stress, and decreased by 33% under severe stress as compared to the control (Fig. 1). The photochemical efficiency (*Fv/Fm*) and net photosynthetic rate (P_n_) of plants were significantly reduced as the water stress increased. The stomatal conductance (g_s_) and transpiration rate (E) also decreased by 34%, 69%, and 83%, and 77%, 82%, and 88% as compared to the control after 3, 7, and 9 days, respectively. However, the intercellular CO_2_ concentration (*C*_i_) increased 41%, 87%, and 169% with increased water stress (mild, moderate, and severe). We also detected the efficiency of open centers (*Fv*′/*Fm*′) and effective quantum yield of PSII (ΦPSII) during the imposed stress, and both decreased as water stress increased (Fig. 2).

### Alteration of plant hormone contents under water stress

**Figure 3:**
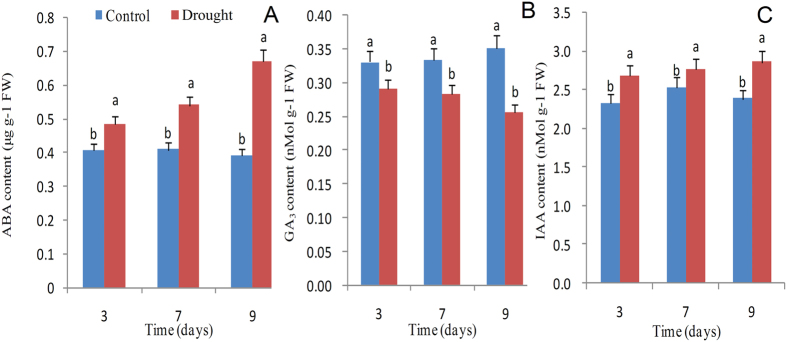
Effects of drought stress on different endogenous hormones ABA (**A**), GA_3_ (**B**) and IAA (**C**) in sugarcane plants. Drought-stressed plants were collected on the 3^rd^, 7^th^, and 9^th^ days, which corresponded to mild, moderate, and severe water stress conditions, respectively. Bars with different superscript letters indicate significant differences at the P < 0.05 probability level at the same treatment time.

The effects of drought stress on endogenous hormones in sugarcane plants were assessed, and results are illustrated in Fig. 3. The ABA and IAA contents showed increases of 20%, 32%, and 72% and 15%, 9%, and 19% under mild, moderate, and severe water stress conditions, respectively. However, the GA_3_ content showed reductions of 12%, 15%, and 27% as compared to the control, respectively.

### Identification of differentially expressed genes

**Figure 4:**
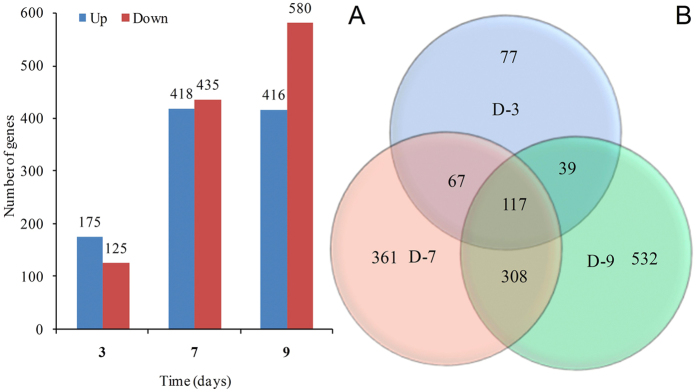
Differential gene expression profiles. (**A**) Numbers of induced and repressed genes under mild, moderate, and severe stress conditions. Up and down indicate up- and downregulated genes. (**B**) Venn diagram presents the number of overlapping genes between the analyzed time points. D-3, D-7 and D-9 represent samples collected on the 3^rd^, 7^th^ and 9^th^ days, which corresponded to mild, moderate, and severe water stress conditions, respectively.

The expression profile of 15,593 genes was evaluated, and 1,501 (9.6%) genes were differentially expressed under drought stress. Among the differentially expressed genes, 821 were upregulated (fold change, FC ≥ 2) and 680 were downregulated (FC ≤ 0.5). Based on the expression profiles for different stress levels, the differentially expressed genes increased as water stress increased. Additionally, the differentially expressed genes appeared to be preferentially induced under mild water stress conditions (3^rd^ d), as shown in Fig. 4A; 175 genes were induced, but only 125 were repressed under this condition. By contrast, genes were preferentially repressed under severe water stress conditions (9^th^ d); 416 genes were induced and 580 were repressed. It is important to emphasize that the expression of many genes overlapped between the three stress levels. The numbers of exclusive and overlapping genes for the different treatment durations under water-deficit conditions are summarized in Fig. 4B.

### Annotation of differentially expressed genes

**Figure 5:**
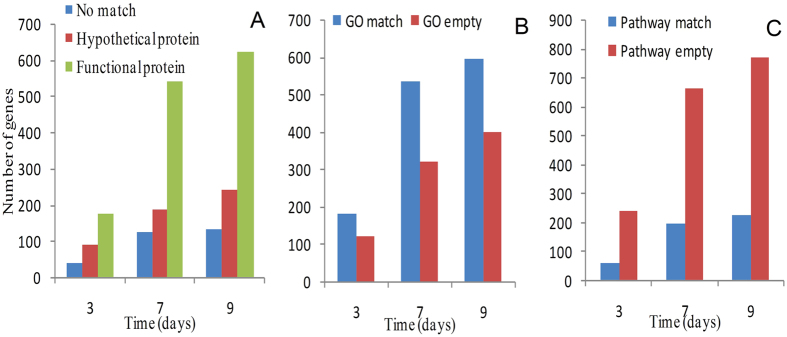
Annotation of differentially expressed genes under water stress. The number of annotated stress-responsive genes is shown for each time point. Genes were successively annotated using the BlastX tool (**A**), AmiGO analysis (**B**) and KAAS tool (**C**). GO (Gene Ontology).

All differentially expressed genes were searched against the NCBI non-redundant database (http://www.ncbi.nlm.nih.gov) using the BlastX tool to find protein similarity (e-value ≤ 10^−20^ cut-off). On day 3 of water stress, 37 of the differentially expressed genes did not demonstrate similarity to genes encoding known proteins, 89 genes had similarity with genes encoding hypothetical proteins in the database, and the remaining 174 genes had similarity with genes encoding functional proteins (Fig. 5A). On days 7 and 9 of water stress, the numbers of genes in the three annotated groups, i.e., those with no similarity, those with similarity with hypothetical proteins, and those with similarity with functional proteins, were 126, 186, and 541 and 132, 241, and 621, respectively (Fig. 5A). The GO annotation results from the AmiGO analysis indicated that 181, 533, and 596 genes had GO term matches in the database on days 3, 7, and 9 of water stress, which represented 60.3%, 62.5%, and 59.8% of the differentially expressed genes, respectively (Fig. 5B). In addition, 60, 193, and 225 genes had pathway matches on days 3, 7, and 9 of water stress, which represented 20.0%, 22.6%, and 22.5% of the differentially expressed genes, respectively (Fig. 5C). By contrast, the number of pathway matches was significantly less than the number of GO term matches.

**Figure 6:**
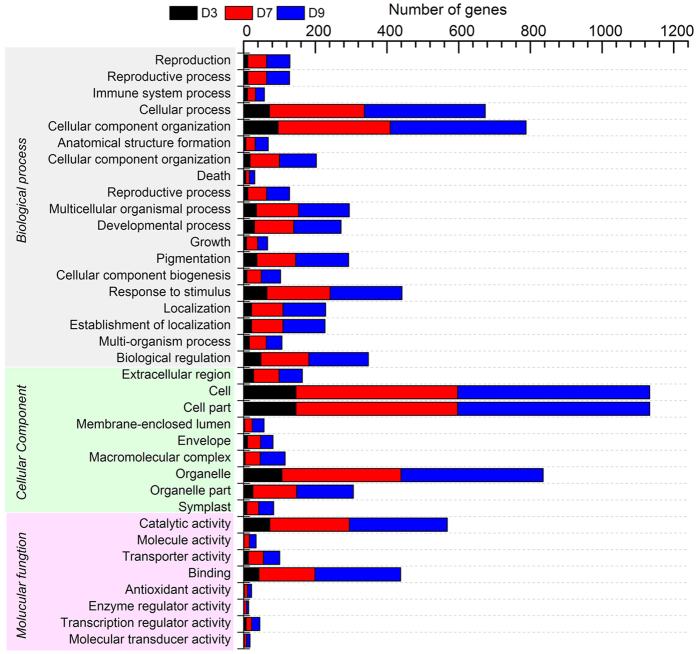
Gene Ontology (GO) classification of differentially expressed genes under water stress. This subset of GO terms was processed by WEGO to categorize the genes into functional groups as follows: biological process, cellular component, and molecular function. D3, D7, and D9 indicated differentially expressed genes at days 3, 7 and 9 under drought stress, respectively.

The GO annotations for differentially expressed genes were submitted to Web Gene Ontology Annotation Plot (WEGO) for GO classification. A total of 901 differentially expressed genes were assigned to 36 GO categories (Fig. 6). Among them, 19, 9, and 8 categories belonged to biological process, cellular components, and molecular functions, respectively. In the biological process category, cellular component organization, cellular process, and response to stimulus comprised the top three terms with respect to the number of differentially expressed genes. For cellular components, the cell, cell part, and organelle categories were the top three with respect to the number of genes. The differentially expressed genes were primarily involved in catalytic activity, molecule activity, transporter activity, binding, antioxidant activity, enzyme regulator activity, transcription regulator activity, and molecular transducer activity.

### Pathway analysis of differentially expressed genes under water stress

**Figure 7:**
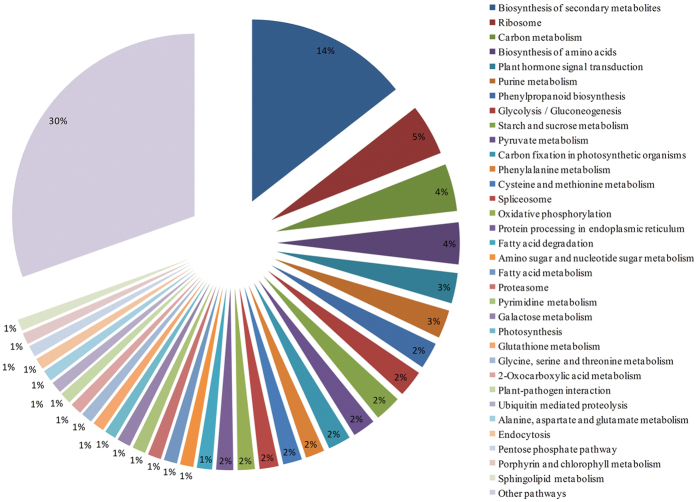
Functional classification of pathway terms for differentially expressed genes under drought stress. The pathway annotations were acquired with KAAS using an e-value threshold of ≤10^−10^. A total of 325 differentially expressed genes were classified into 101 pathway categories. The data for pathway categories that represented less than 1% of the differentially expressed genes were included in other pathway categories.

The 325 nonoverlapping differentially expressed genes were classified into 101 pathway categories by KAAS with an e-value threshold of ≤10^−10^. The most highly represented pathway slim categories were biosynthesis of secondary metabolites (14%), ribosome (5%), carbon metabolism (4%), and biosynthesis of amino acids (4%), suggesting a high degree of basic metabolic activity in the regulation of gene expression under water stress conditions (Fig. 7). The plant hormone signal transduction (3%) category occupied 5^th^ place, indicating an important role of plant hormones in responses to water deficits. The overrepresented stress-induced genes were broadly classified into purine metabolism (3%), phenylpropanoid biosynthesis (2%), glycolysis /gluconeogenesis (2%), starch and sucrose metabolism (2%), pyruvate metabolism (2%), carbon fixation in photosynthetic organisms (2%), phenylalanine (2%), cysteine and methionine metabolism (2%), and spliceosome (2%). The genes in each pathway category are shown in Supplementary Table S1.

### Validation of microarray results using quantitative real-time PCR

To validate the microarray results, 15 differentially expressed genes were selected and analyzed by real-time PCR. For each gene, three biological replicates were assayed in duplicate using the same RNA samples as those used in the microarray experiment. The amplification specificity of the primers was evaluated based on melting curves generated from qPCR runs. Negative controls were used to confirm the absence of contamination. The expression of the positive control in our samples was considered stable according to the amplification curves obtained in the assays. The relative quantification of target genes was calculated using the 2^−ΔΔCt^ method. The expression levels of selected genes in the qPCR analysis are summarized in Supplementary Table S2. The sugarcane genes assayed by qPCR were differentially expressed under water stress conditions, and the FC of gene expression profiles were very similar to those observed in the microarray experiments. These results indicated that the microarray experiments in this study are sufficiently reliable for the identification of genes involved in water deficit responses in sugarcane.

## Discussion

Drought stress tolerance in plants is influenced by the developmental stage, organ or tissue type, and exposure duration and severity^21^. Low water levels in the soil elicit a range of responses that allow plants to avoid or tolerate water loss through physiological, biochemical, and molecular processes^21^. Physiological modifications are the first responses against water deficits, and photosynthesis is the major process affected by drought^3^. Water stress results in photo-inhibition, which includes photo-damage to the photosynthetic apparatus and causes irreversible inactivation of PSII, as evidenced by remarkable changes in photosynthetic parameters. In this study, alterations in the net photosynthetic rate (P_n_), stomatal conductance (g_s_), intercellular CO_2_ concentration (C_i_), and transpiration rate (E) were assessed under progressive water stress conditions at the elongation stage of sugarcane plants. Photosynthetic efficiency was significantly reduced by water stress, which was in strong agreement with the results of previous studies in other plants species, such as sugarcane^22^, eucalyptus^23^, wheat^24^, rice^25^, and tobacco^26^. Chlorophyll a fluorescence is normally accepted as a method to determine the structure and function of the photosynthetic apparatus^27^. The analysis of chlorophyll a fluorescence is considered important to evaluate the health and integrity of the internal apparatus during photosynthesis. In this study, the maximum quantum yield (*Fv/Fm*), effective quantum yield of PSII (ΦPSII), and capture efficiency of open centers (*Fv*′/*Fm*′) reflected the severity of drought stress, and similar results have been also reported in rice^28^, watermelon^29^, and wheat^30^.

The effects of water stress on photosynthesis include the restriction of CO_2_ diffusion into the chloroplast, limitations on stomatal opening, and mesophyll transport of CO_2_ mediated by plant hormones whose biosynthesis, accumulation, and redistribution lead to alterations in leaf photochemistry and carbon metabolism^6^. Of the plant hormones, ABA plays a central role in controlling the stomatal aperture^9^. Drought triggers the production of ABA in roots or leaves, which is transported to the shoots, causing stomatal closure, decreasing transpiration rate, and eventually restricting cellular growth^9^. Other reports show that stomatal function also depends on other hormones and the degree of their interactions^31^. In this study, we investigated changes in three plant hormones in sugarcane under water-stressed environments. The ABA and IAA contents increased dramatically, but GA_3_ declined rapidly in leaves from the beginning to the end of drought treatment. Generally, auxin, cytokinin, and ethylene tend to inhibit ABA-mediated stomatal closure, where as jasmonates, brassinosteroids, and salicylic acid display a concurrent action with ABA^31^, both ABA and IAA are involved in the development of defensive responses during adaptation to drought^9^. However, the complex regulatory network of phytohormone signaling in plants subjected to water stress needs to be examined in more detail.

Gene expression alterations can promote cellular adaptation to water stress and gene expression profiles reveal the complexity of defense mechanisms^8^. Microarrays are a powerful tool for identifying differentially expressed genes owing to their high-throughput property, which permits the simultaneous analysis of many genes. In this study, the expression patterns obtained by microarray for genes encoding phosphatase, protein kinase, phytochrome, sucrose synthase, starch synthase, beta-glucosidase, and other proteins (Supplementary Table S2) were analyzed by real-time PCR. The results revealed significant correlations, indicating that the microarray was efficient for the identification of genes expressed in sugarcane leaves under water deficit conditions. In this study, 15,593 sugarcane genes were monitored, but only 9.6% of the genes (n = 1,501) were differentially expressed under water stress. Functional annotation analyses of genes that are differentially expressed in response to water stress have been conducted in several previous studies^18^,^20^. These reports described the expression and regulation profiles of many genes, but the majority of identified genes encode unknown proteins or genes without sequence similarity in databases. In the present study, 42%, 36.6%, and 37.6% of the differentially expressed genes on days 3, 7, and 9 of water stress showed unknown functions (no match + hypothetical protein). GO and pathway annotation results also revealed that many genes had no similarity in the databases, which was similar to previous results in maize^32^. Gorantla *et al*.^33^ identified differentially expressed genes in rice plants grown under drought conditions and found that about 43% of the unigenes represented functionally unknown genes, such as putative, predicted, hypothetical, or unknown proteins. The characterization of undescribed genes is important for research on water stress tolerance because they may represent new sources of variability and may contribute to our understanding of the complex interactions between gene expression and physiological modifications involved in the plant water stress response network.

Water stress induces physiological changes that genuinely affect plant metabolism. One of the major plant molecular responses against immediate dehydration is to activate protective mechanisms via gene expression alteration related to different pathways^8^. Secondary metabolites also play a very important role in the adaptation or protection of plants to environmental stress^34^. In our analysis, 86 genes were involved in the biosynthesis of secondary metabolites, which was the largest pathway category of the differentially expressed genes. Among them, the expression levels of seven genes encoding peroxidases (CA175216, CA273769, CA279133, CA274665, CA146051, CA184143, and CA205715) were altered under drought stress. Peroxidases have been implicated in defense against pathogens and detoxification metabolism^22^. The detoxification process is very important to stress events due to reactive oxygen species produced in adverse conditions, such as hydrogen peroxide scavenged by peroxidase^22^. The expression levels of three genes involved in chlorophyll metabolism that encode magnesium-protoporphyrin IX monomethyl ester cyclase (CA294821), pheophorbide A oxygenase (CA286120), and chlorophyllide A oxygenase (CA088276) were all downregulated as compared to the control. By contrast, the genes BU103703 and CA230914, encoding delta-1-pyrroline-5-carboxylate synthetase and 9-*cis*-epoxycarotenoid dioxygenase, which are the key determinants of plant endogenous biosynthesis of ABA and proline, were upregulated by more than 4-fold under water stress, consistent with the increases in ABA and proline production observed previously under drought conditions^35^. A previous study indicated that a wide variety of secondary metabolites are synthesized from primary metabolites, such as carbohydrates, lipids, and amino acids, in higher plants^36^. In this study, 22 differentially expressed genes participated in the biosynthesis of the amino acid pathway (Supplementary Table S1), indicating that different amino acids represent functional diversity under water stress. The ribosomes are an intricate ribonucleoprotein complex with multiple protein constituents in equimolar amounts, and the coordination of the synthesis of these ribosomal proteins presents a major challenge to the plant cell. Many ribosomal proteins were also found in this study (n = 27). McIntosh and Bonham-Smith^37^ discussed the alteration in plant ribosomal protein gene expression under salinity, cold, water stress, plant hormone treatment, or stress-associated events and suggested a function in restoring the protein synthesis processes.

Water stress can severely reduce the productivity of sugarcane by inhibiting the photosynthetic rate^21^,^38^. At the first sign of water deficit, plants close the stomata to avoid excessive water loss by transpiration and as a consequence, under moderate water stress, photosynthesis is affected and is eventually inhibited by increasing water stress severity^38^. Nine differentially expressed genes were identified in the photosynthesis pathway in this study. Other than the genes encoding photosystem II 10kDa protein (CA181846) and light-harvesting complex I chlorophyll a/b binding protein 2 (CA110540), which were induced, the other seven genes were deeply repressed by drought conditions, which was in accordance with the physiological data showing that the photosynthetic rate was progressively decreased during water stress. The relationship between water in sugarcane and the plant response to water deficits was also confirmed by Inman-Bamber and Smith^2^; they reported that the net photosynthetic rate and consequent productivity depend on the status of the metabolic process in cells during stress. Only under severe stress do the pyruvate orthophosphate dikinase activity and pyruvate levels become decreased. In this study, the gene encoding pyruvate orthophosphate dikinase (CA191099) was sharply repressed by severe water stress. However, both the enzymes phosphoenolpyruvate carboxylase (CA196624) and carbonic anhydrase (CA123382) were highly induced under stress. Among the genes involved in the pyruvate metabolism pathway, pyruvate kinase (CA251534, CA228227, and CA189611) was induced with a greater than 2-fold change; pyruvate kinase participates in glycolysis and is very important for the regulation of this pathway under stress conditions^39^. Soluble sugars play an important role in plant metabolism as sources of carbon and energy in cells, and the continual adjustment between the supply and utilization of carbon, sucrose-starch partition is necessary for plants under stress conditions^40^. Sugars are also important signaling molecules for photosynthesis. It has been proposed that intracellular sugar concentrations exert a feedback control on the rate of photosynthesis, and these feedback mechanisms lead to significant changes in enzyme activity and gene expression^41^. In this study, 25, 14, and 7 genes were found to be involved in carbon metabolism, starch and sucrose metabolism, and the amino sugar and nucleotide sugar metabolism pathway, and the expression patterns of these genes showed huge diversity depending on the degree of stress. The alteration in the magnitude of the sugar pools is determined by changes in the activity of enzymes involved in sugar- and starch-related pathways, such as starch synthase (CA258249), sucrose synthase (CA175853 and CA196779), and glucosidase (CA103087, CA284699, CA134312, and CA217026). Although these enzymes have been identified in various abiotic stress studies^3^, their associations with drought and photosynthesis are not fully understood and need to be examined with respect to their roles in stress responses.

The plant response begins with stress recognition at the cellular level via the activation of signal transduction pathways. In our study, 16 genes were involved in plant hormone signal transduction pathways. Previous studies have shown that a pathway based on PYR/RCAR ABA receptors, protein phosphatase 2C (PP2Cs), and serine/threonine-protein kinase 2 (SnRK2s) forms the primary basis of an early ABA signaling module. Generally, the PYR/RCARs act as ABA receptors, the PP2Cs act as negative regulators of the pathway, and SnRK2s act as positive regulators of downstream signaling^42^,^43^. Under normal conditions, in the absence of ABA, pre-existing PP2Cs appear to repress the ABA signaling pathways *via* the inactivation of SnRK2s. Under water stress conditions, as the production of ABA increases, ABA-bound PYR/RCARs interact with PP2Cs and inhibit phosphatase activity, promoting SnRK2 activation and the phosphorylation of target proteins^43^,^44^. In this study, CA246148 (PP2Cs) was repressed (potentially reflecting gene redundancy), but the expression of other PP2Cs (CA196644, CA078060, and CA093454) and SnRK2 (CA129160 and CA280103) were significantly induced by water stress and were correlated with the maximum enzyme activity under water stress conditions^42^,^43^. The ABA-responsive element binding factor (CA095141) genes were also a key component, as they are activated by SnRK2s in this transduction pathway^9^. The expression of BR-signaling kinase (CA285332), a member of the BSK family that activates brassinosteroid signaling downstream of BRI1^45^, three cytokinin response regulators (CA113863, CA154411, and CA191067), which are negative regulators in cytokinin signaling^46^, and coronatine-insensitive protein (CA133645), the primary jasmonic acid receptor^47^, were altered under drought conditions. A previous study revealed that the coronatine-insensitive protein was important in signaling interactions between ABA and methyl jasmonate in plant guard cells, specific impairment of ion channel activation, and second messenger production^45^,^48^.

In summary, the results of this study revealed the gene expression alterations in sugarcane exposed to progressive drought conditions as well as their possible pathways. Sugarcane responses depended mainly on the early perception of stress, and the activation of downstream genes, which included signaling molecules and genes regulated by plant hormones or important for protection against oxidative stress. Great progress has been made in recent years to elucidate the nature of various factors affecting plant metabolism in response to water deficits and signaling cascades that link and coordinate the regulation of the metabolic pathways acting in parallel to provide necessary tolerance phenotypes under stress. Moreover, the high number of differentially expressed genes with unknown function is intriguing. These genes need to be further investigated to determine their functional importance in order to improve our knowledge of drought tolerance in sugarcane plants. Elucidating the mechanisms of plant tolerance to drought responses would assist in plant molecular breeding to develop drought-resistant varieties.

## Materials and Methods

### Plant growth and water stress treatment

The experiment was conducted in a completely randomized block design using pot (diameter 30 cm; height 35 cm) culture. Single bud sets of sugarcane cultivar GT 21 were initially raised by standard culture techniques, the 50-day-old settlings were transplanted into the pots containing 17.5 kg soil mix [clay soil/organic manure/sand, 70:20:10 ratio, w/w)] with a basal dose of NPK fertilizer (26 g N + 1.76 g P + 20 g K pot^−1^) in greenhouse conditions. Urea (46.4% N), phosphate (12% P_2_O_5_) and potassium oxide (60% K_2_O) fertilizer were used as the source of N, P and K, respectively. The pots were irrigated with water every day. The drought treatment was given at the 5 month growth stage of the plants. These tillering and grand growth stages, known as the sugarcane formative phase, have been identified as the critical water demand period^49^, mainly because this is the phase when 70–80% of cane yield is produced^50^. The drought treatment in pots was maintained by withholding the water during the course of experiment (9 days). The total moisture content was measured with gravimetric method, i.e. % soil moisture (dw) = 100 × [(Fresh weight − dry weight)/dry weight]. The total moisture content in control and drought treated pots were maintained at 20 ± 2% and 9 ± 2%, respectively. The water-stressed samples collected on the 3^rd^, 7^th^ and 9^th^ days of the experimental treatment were designated as mild, moderate and severe water deficit conditions, respectively. After photosynthesis and chlorophyll fluorescence measurements, the top visible dewlap leaves samples were detached from five sugarcane plants for each treatment and frozen in liquid nitrogen for physiological, biochemical parameters and microarray analyzing, all samples were analyzed in triplicate. Leaf relative water content (RWC) was measured gravimetrically as described by Silva *et al*.^51^.

### Hormone extraction and measurement

A total of 1 g leaf sample was ground with an iced mortar and pestle in 5 mL of 80% (v/v) methanol extraction containing 1 mM butylated hydroxytoluene as an antioxidant. The homogenate was kept at 4 °C for 12 h, centrifuged at 10,000 × *g* for 20 min, and the supernatant was collected. The residue was extracted again at 4 °C for 1 h, centrifuged, and the supernatants were combined and the volume was recorded. The extracted solution was eluted through a Sep-Pak C18 cartridge (Waters, Milford, MA, USA) to remove the pigment.The solution was dried in a freezing dryer (Labconco, England), and dissolved with 2 mL of phosphate-buffered saline containing 0.1% (v/v) Tween 20 and 0.1% (w/v) gelatin (pH 7.5). Contents of abscisic acid (ABA), indole-3-acetic acid (IAA) and gibberellin (GA_3_) were determined with an enzyme-linked immunosorbent assay described by Yang *et al*.^52^ with a 96-well microtiter plate that was pre-coated with coating buffer containing synthetic ovalbumin conjugates for IAA, GA3, and ABA by the manufacturer (China Agricultural University, Beijing, China). Fifty microliters of standard or sample in assay buffer (1,000 mL of H_2_O containing 8.0 g of NaCl, 0.2 g of KH_2_PO_4_, 2.96 g of Na_2_HPO_4_·12H_2_O, 1.0 mL of Tween-20, and 1.0 g of gelatin) was added to each well, followed by 50 μL of antibodies diluted (1:2,000) in assay buffer. The plates were incubated for 45 min at 37 °C and then washed four times with scrubbing buffer (the same buffer as the assay buffer, without gelatin). Anti-mouse IgG coupled to alkaline phosphatase (100 μL of a 1:1,000 dilution) was added to each well. The plates were incubated for 30 min at 37 °C and then washed as above. Next, 100 μL of a 1.5 mg mL^−1^ ortho-phenylenediamine substrate solution and 0.04% (v/v) of 30% H_2_O_2_ in substrate buffer (1,000 mL of H_2_O containing 5.10 g of C_6_H_8_O_7_·H_2_O, 18.43 g of Na_2_HPO_4_·12H_2_O, and 1.0 mL of Tween-20) were added. The enzyme reaction was carried out in the dark at 37 °C for 15 min, and then stopped by adding 50 μL of 2 M H_2_SO_4_ per well. The absorbance was read at 490 nm with a Thermo Electron Multiskan MK3 (Pioneer Co., Shenzhen, China). Contents of IAA, GA3, and ABA were calculated from logit *B/B*_*0*_-transformed standard curve data, in which B and B_0_ are the absorbance values in the presence and absence of the competing antigen, respectively.

### Photosynthesis and chlorophyll fluorescence measurements

The net photosynthetic rate (P_n_), stomata conductance (g_s_), intercellular CO_2_ concentration (C_i_), and transpiration rate (E) were measured using the portable photosynthesis system Li-6400 (LI-COR Biosciences, Lincoln, NE, USA) under a photon flux density of 1,000 μmol m^−2^s^−1^. Fluorescence measurements were performed with a modulated fluorometer (FMS-2, Hansatech, Norfolk, UK) using the saturation pulse method. Thirty-min dark-adapted leaves were first exposed to an actinic light and saturated light provided by the fluorometer to obtain the minimum fluorescence (*F*_*0*_) and maximum fluorescence (*F*_*m*_), respectively. Then, a photon flux density of 1,000 μmol m^−2^ s^−1^ was started every 20 s to get the light-adapted fluorescence maximum (*Fm*′), steady-state fluorescence yield (*Fs*), and *Fo*′, which is the minimum fluorescence after far-red illumination of the previously exposed leaves. The following parameters were calculated^53^: the maximum quantum yield of PSII [*Fv*/*Fm* = (*Fm* − *Fo*)/*Fm*], the excitation capture efficiency of open centers [*Fv*′/*Fm*′ = (*Fm*′ − *Fo*′)/*Fm*′], and the effective quantum yield of PSII [ΦPSII = Δ*F*/*Fm*′ = (*Fm*′ − *Fs*)/*Fm*′].

### RNA extraction, microarray hybridization, and data analysis

The Agilent sugarcane oligo microarray (4 × 44 K) containing 15,593 probes were established to represent the same number of genes on a single array. Total RNA was extracted from the leaves using the commercial TRIzol reagent (Invitrogen, Carlsbad, CA, USA) according to the manufacturer’s instructions. The RNA extracts were detected for purity with a Bioanalyzer 2100 (Agilent Technologies, Santa Clara, CA, USA), then amplified, labeled, and purified using a GeneChip 3′ IVT Express Kit (Affymetrix, Santa Clara, CA, USA) to obtain biotin-labeled cRNA. The cRNA labeling, hybridization, and scanning of the microarrays were performed according to Agilent one-channel chip operation procedure by Shanghai Biotech Co. (www.shbiochip.com). The hybridization results were scanned using the GeneChip Scanner 3,000 and normalization per chip of the log2 values was performed with the MAS 5.0 algorithm (Gene Spring Software 11.0, Agilent Technologies) for comparative analyses of the three replicates of the water stress and control treatments for each experiment. The quality control and quality assurance measure was average background <100. A gene with an FC of ≥2.0 or ≤0.5 (up- or down regulation) was defined as a differentially regulated gene, and the FC data were filtered by *t*-tests (P ≤ 0.05). To avoid false positives, the minimum false discovery rate (FDR) was calculated, and the differentially expressed genes were selected at FDR (q) <0.05. The functional annotation of differentially expressed genes were searched against the NCBI protein data base using BLASTX with an E-value ≤ 10^−20^. The GO annotations were searched by an AmiGO analysis (http://amigo1.geneontology.org) (e-value ≤ 10^−10^ cut-off), and the results were plotted using the annotation numbers in the Web Gene Ontology Annotation Plot (WEGO) (http://wego.genomics.org.cn/cgi-bin/wego/index.pl). The pathway annotations were performed with KAAS (http://www.genome.jp/kaas-bin/kaas_main) (e-value ≤ 10^−10^ cut-off).

### Gene expression analysis by qRT-PCR

For expression validation of differentially expressed genes, special primers were designed for 15 differentially expressed genes, randomly selected in different time points using Primer Premier 5.0 software (Supplementary Table S2). The specificity of each primer set was checked by sequencing PCR products and efficiencies of the different primer sets were similar. Total RNA used as template for reverse transcriptase reactions was initially treated with DNase I Amplification Grade enzyme (Invitrogen), an aliquot of treated RNA was used in qPCR to rule out DNA contamination. cDNA synthesis was done using SuperScript First-Strand Synthesis System for RT-PCR (Invitrogen) and random hexamers and oligo(dT) primers. qPCR reaction were performed in a volume of 20 μL containing 10 μL 2 × All-in-One^TM^ (GeneCopoeia, Los Angeles, USA) qPCR Mix, 2 μL cDNA, 1 μL of each 4 μM primer, and 6 μL RNase-free sterile water. To normalize the relative expression of selected genes, GAPDH gene was used as reference. qPCR was performed using in an iQ5 Real Time PCR Detection System (Bio-Rad, Hercules, USA). PCR reactions were performed at 95 °C for 10 min, followed by 40 cycles of 95 °C for 10 s, 60 °C for 20 s and 72 °C for 20 s. Melting curve analysis was conducted for each reaction to confirm the specificity of the reaction, all the cDNA samples were analyzed in triplicate. Relative expression levels of candidate genes were calculated using 2^−ΔΔCt^ method^54^.

## Additional Information

**How to cite this article**: Li, C. *et al*. Differential expression profiles and pathways of genes in sugarcane leaf at elongation stage in response to drought stress. *Sci. Rep*. **6**, 25698; doi: 10.1038/srep25698 (2016).

## Supplementary Material

Supplementary Information
