# Supplementary material for: Differential expression profiles and pathways of genes in sugarcane leaf at elongation stage in response to drought stress
Source: Sci Rep. 2016 May 12;6:25698. doi: 10.1038/srep25698 (PMC4864372; doi:10.1038/srep25698)
Supplement: Supplementary Information [file srep25698-s1.pdf]

# Differential expression profiles and pathways of genes in sugarcane leaf at elongation stage in response to drought stress

Changning Li[1], Qian Nong[3], Manoj Kumar Solanki[1], Qiang Liang[1], Jinlan Xie[1], Xiaoyan Liu[1], Yijie Li[1], Weizan Wang[1], Litao Yang[2]* and Yangrui Li[1]*

[1]Key Laboratory of Sugarcane Biotechnology and Genetic Improvement (Guangxi), Ministry of Agriculture, Guangxi Key Laboratory of Sugarcane Genetic Improvement, Sugarcane Research Center of Chinese Academy of Agricultural Sciences, Sugarcane Research Institute of Guangxi Academy of Agricultural Sciences, Nanning, Guangxi 530007, China. [2]College of Agriculture, State Key Laboratory of Conservation and Utilization of Subtropical Agro-bioresources, Guangxi University, Nanning, Guangxi 530004, China. [3]Microbiology Research Institute of Guangxi Academy of Agricultural Sciences, Nanning, Guangxi 530007, China.

Correspondence and requests for materials should be addressed to L. Y. (email: liyr@gxu.edu.cn) or Y. L. (email: liyr@gxaas.net; liyr5745@126.com)

Supplementary information

Table S1 Pathway category details of stress-responsive genes

Table S2 Validation of selected candidate genes using real time-PCR

Table S1 Pathway category details of stress-responsive genes

| Access GenBank | Description | Blast species | e_value | D3_FC | D7_FC | D9_FC |
|---|---|---|---|---|---|---|
| **Photosynthesis** | | | | | | |
| CA285765 | ATPF1G; F-type H+-transporting ATPase subunit gamma | Zea mays | 4.0E-110 | — | 0.198 | 0.202 |
| CA272525 | petE; plastocyanin | Zea mays | 2.0E-56 | 0.399 | 0.152 | — |
| CA216892 | petH; ferredoxin--NADP+ reductase [EC:1.18.1.2] | Zea mays | 2.0E-86 | 0.472 | — | — |
| CA125184 | psaB; photosystem I P700 chlorophyll a apoprotein A2 | Oryza sativa | 2.0E-134 | — | 0.375 | 0.212 |
| CA297237 | psbM; photosystem II PsbM protein | Oryza rufipogon | 4.0E-26 | — | 0.408 | — |
| CA181846 | psbR; photosystem II 10kDa protein | Zea mays | 3.0E-102 | — | — | 2.236 |
| CA110540 | LHCA2; light-harvesting complex I chlorophyll a/b binding protein 2 | Setaria italica | 0.0E+00 | 4.920 | 5.832 | — |
| CA180915 | LHCA4; light-harvesting complex I chlorophyll a/b binding protein 4 | Zea mays | 2.0E-111 | — | 0.431 | — |
| CA276082 | LHCB1; light-harvesting complex II chlorophyll a/b binding protein 1 | Zea mays | 2.0E-131 | — | — | 0.079 |
| **Biosynthesis of secondary metabolites** | | | | | | |
| CA185994 | GPD1; glycerol-3-phosphate dehydrogenase (NAD+) [EC:1.1.1.8] | Zea mays | 2.0E-155 | — | 2.658 | 2.538 |
| CA206172 | MDH2; malate dehydrogenase [EC:1.1.1.37] | Zea mays | 0.0E+00 | — | — | 3.576 |
| CA143081 | cinnamyl-alcohol dehydrogenase [EC:1.1.1.195] | Setaria italica | 7.0E-85 | — | — | 0.375 |
| CA110802 | cinnamyl-alcohol dehydrogenase [EC:1.1.1.195] | Zea mays | 7.0E-115 | — | 36.961 | 40.257 |
| CA121038 | cinnamyl-alcohol dehydrogenase [EC:1.1.1.195] | Zea mays | 2.0E-103 | — | 0.442 | — |
| CA226469 | frmA; S-(hydroxymethyl)glutathione dehydrogenase / alcohol dehydrogenase [EC:1.1.1.284 1.1.1.1] | Zea mays | 6.0E-119 | — | 0.389 | — |
| CA160929 | PPOX; oxygen-dependent protoporphyrinogen oxidase [EC:1.3.3.4] | Zea mays | 3.0E-86 | 2.979 | 2.502 | 2.267 |
| CA261850 | acyl-CoA oxidase [EC:1.3.3.6] | Zea mays | 3.0E-97 | — | 0.250 | 0.224 |
| CA192696 | AOC3; primary-amine oxidase [EC:1.4.3.21] | Setaria italica | 6.0E-114 | — | — | 2.050 |
| CA120928 | DLD; dihydrolipoamide dehydrogenase [EC:1.8.1.4] | Zea mays | 0.0E+00 | — | — | 2.573 |
| CA275091 | polyphenol oxidase [EC:1.10.3.1] | Sorghum bicolor | 0.0E+00 | — | — | 47.878 |
| CA175216 | peroxidase [EC:1.11.1.7] | Sorghum bicolor | 0.0E+00 | — | — | 2.718 |
| CA273769 | peroxidase [EC:1.11.1.7] | Setaria italica | 6.0E-88 | — | 0.204 | 0.201 |
| CA279133 | peroxidase [EC:1.11.1.7] | Zea mays | 0.0E+00 | — | — | 0.487 |
| CA274665 | peroxidase [EC:1.11.1.7] | Zea mays | 4.0E-131 | 0.334 | — | 0.364 |
| CA146051 | peroxidase [EC:1.11.1.7] | Zea mays | 4.0E-131 | 0.334 | — | 0.364 |

Table S1 (Continued)

| Access GenBank | Description | Blast species | e_value | D3_FC | D7_FC | D9_FC |
|---|---|---|---|---|---|---|
| CA184143 | peroxidase [EC:1.11.1.7] | Zea mays | 9.0E-134 | — | 0.390 | 0.352 |
| CA205715 | peroxidase [EC:1.11.1.7] | Setaria italica | 9.0E-99 | — | 5.305 | 3.047 |
| CA065868 | caffeoyl-CoA O-methyltransferase [EC:2.1.1.104] | Zea mays | 4.0E-69 | 28.751 | — | 18.526 |
| CA206289 | purH; phosphoribosylaminoimidazolecarboxamide formyltransferase / IMP cyclohydrolase [EC:2.1.2.3 3.5.4.10] | Saccharum hybrid cultivar | 3.1E-155 | 2.199 | — | — |
| CA280616 | acetyl-CoA C-acetyltransferase [EC:2.3.1.9] | Zea mays | 1.0E-161 | — | — | 3.742 |
| CA103835 | DLAT; pyruvate dehydrogenase E2 component (dihydrolipoamide acetyltransferase) [EC:2.3.1.12] | Zea mays | 0.0E+00 | — | — | 3.916 |
| CA094588 | cysE; serine O-acetyltransferase [EC:2.3.1.30] | Setaria italica | 2.0E-97 | — | 3.230 | 3.343 |
| CA113654 | CHS; chalcone synthase [EC:2.3.1.74] | Brachypodium distachyon | 1.0E-114 | — | 0.190 | 0.115 |
| CA258249 | starch synthase [EC:2.4.1.21] | Zea mays | 5.0E-36 | 0.483 | 0.429 | 0.474 |
| CA184092 | metK; S-adenosylmethionine synthetase [EC:2.5.1.6] | Zea mays | 1.0E-114 | — | — | 0.067 |
| CA228575 | TAT; tyrosine aminotransferase [EC:2.6.1.5] | Setaria italica | 0.0E+00 | — | — | 2.547 |
| CA096192 | AGXT2; alanine-glyoxylate transaminase/(R)-3-amino-2-methylpropionate-pyruvate transaminase [EC:2.6.1.44 2.6.1.40] | Zea mays | 1.0E-73 | 6.685 | 7.437 | 4.073 |
| CA140452 | AGXT2; alanine-glyoxylate transaminase/(R)-3-amino-2-methylpropionate-pyruvate transaminase [EC:2.6.1.44 2.6.1.40] | Zea mays | 4.0E-25 | 3.693 | 5.295 | 2.246 |
| CA214701 | pfkA; 6-phosphofructokinase 1 [EC:2.7.1.11] | Zea mays | 3.0E-65 | — | — | 0.285 |
| CA189611 | PK; pyruvate kinase [EC:2.7.1.40] | Brachypodium distachyon | 2.0E-161 | — | 2.385 | 3.140 |
| CA228227 | PK; pyruvate kinase [EC:2.7.1.40] | Zea mays | 1.0E-110 | 2.158 | 3.444 | 2.180 |
| CA251534 | PK; pyruvate kinase [EC:2.7.1.40] | Zea mays | 9.0E-89 | 3.466 | — | — |
| CA234341 | dgkA; diacylglycerol kinase (ATP) [EC:2.7.1.107] | | | — | 0.458 | — |
| CA206652 | PGK; phosphoglycerate kinase [EC:2.7.2.3] | Setaria italica | 3.0E-107 | — | 3.624 | — |
| CA219909 | lysC; aspartate kinase [EC:2.7.2.4] | Zea mays | 8.0E-122 | 2.210 | 2.839 | 3.104 |
| CA124145 | ndk; nucleoside-diphosphate kinase [EC:2.7.4.6] | Zea mays | 2.0E-142 | — | — | 0.399 |
| CA224931 | PGLS; 6-phosphogluconolactonase [EC:3.1.1.31] | Zea mays | 1.0E-92 | 2.186 | 2.791 | — |
| CA071439 | PLD1_2; phospholipase D1/2 [EC:3.1.4.4] | Zea mays | 2.0E-32 | — | 0.456 | — |

Table S1 (Continued)

| Access GenBank | Description | Blast species | e_value | D3_FC | D7_FC | D9_FC |
|---|---|---|---|---|---|---|
| CA103087 | beta-glucosidase [EC:3.2.1.21] | Setaria italica | 8.0E-160 | — | — | 0.052 |
| CA284699 | beta-glucosidase [EC:3.2.1.21] | Oryza sativa | 9.0E-132 | 0.266 | 0.355 | 0.327 |
| CA238858 | phosphoenolpyruvate carboxykinase (ATP) [EC:4.1.1.49] | Thyridolepis mitchelliana | 7.0E-164 | — | 0.218 | 0.145 |
| CA228285 | acetolactate synthase I/II/III large subunit [EC:2.2.1.6] | Zea mays | 1.0E-88 | 2.143 | 2.261 | 2.375 |
| CA234827 | acetolactate synthase I/III small subunit [EC:2.2.1.6] | Zea mays | 2.0E-135 | — | 0.264 | 0.138 |
| CA264504 | ACO; aconitate hydratase [EC:4.2.1.3] | Pinus pinaster | 4.0E-151 | — | 6.909 | 5.462 |
| CA134028 | cysK; cysteine synthase A [EC:2.5.1.47] | Zea mays | 2.0E-113 | — | 0.259 | 0.194 |
| CA224791 | metC; cystathionine beta-lyase [EC:4.4.1.8] | Setaria italica | 0.0E+00 | — | — | 0.450 |
| CA100020 | hemH; ferrochelatase [EC:4.99.1.1] | Sorghum bicolor | 0.0E+00 | — | — | 0.399 |
| CA261190 | galM; aldose 1-epimerase [EC:5.1.3.3] | Sorghum bicolor | 0.0E+00 | 0.415 | — | — |
| CA289954 | glucose-6-phosphate 1-epimerase [EC:5.1.3.15] | Setaria italica | 7.0E-134 | — | 0.269 | 0.302 |
| CA134928 | rpiA; ribose 5-phosphate isomerase A [EC:5.3.1.6] | Zea mays | 3.0E-86 | 0.479 | — | — |
| CA285944 | rpiA; ribose 5-phosphate isomerase A [EC:5.3.1.6] | Zea mays | 5.0E-91 | — | 0.366 | — |
| CA165609 | GPI; glucose-6-phosphate isomerase [EC:5.3.1.9] | Zea mays | 9.0E-154 | — | 0.257 | 0.189 |
| CA272115 | GPI; glucose-6-phosphate isomerase [EC:5.3.1.9] | Zea mays | 6.0E-131 | — | 0.215 | 0.296 |
| CA151646 | EARS; glutamyl-tRNA synthetase [EC:6.1.1.17] | Setaria italica | 7.0E-148 | — | 0.245 | — |
| CA243497 | purM; phosphoribosylformylglycinamidine cyclo-ligase [EC:6.3.3.1] | Setaria italica | 8.0E-81 | — | 0.383 | 0.375 |
| CA221482 | purL; phosphoribosylformylglycinamidine synthase [EC:6.3.5.3] | Oryza sativa | 7.0E-132 | — | 0.497 | — |
| CA265748 | asnB; asparagine synthase (glutamine-hydrolysing) [EC:6.3.5.4] | Zea mays | 3.0E-125 | — | 0.353 | 0.223 |
| CA134623 | asnB; asparagine synthase (glutamine-hydrolysing) [EC:6.3.5.4] | Zea mays | 3.0E-141 | — | 10.266 | — |
| CA177243 | wrbA; NAD(P)H dehydrogenase (quinone) [EC:1.6.5.2] | Setaria italica | 0.0E+00 | — | — | 0.461 |
| CA294821 | magnesium-protoporphyrin IX monomethyl ester (oxidative) cyclase [EC:1.14.13.81] | Setaria italica | 2.0E-179 | 0.369 | — | — |
| CA223665 | ent-copalyl diphosphate synthase [EC:5.5.1.13] | Zea mays | 5.0E-149 | — | 0.058 | 0.032 |
| CA186391 | GA3; ent-kaurene oxidase [EC:1.14.13.78] | Zea mays | 3.0E-35 | — | 2.035 | — |
| CA134312 | bglB; beta-glucosidase [EC:3.2.1.21] | Zea mays | 7.0E-139 | 3.956 | 2.782 | 5.845 |
| CA217026 | bglB; beta-glucosidase [EC:3.2.1.21] | Setaria italica | 9.0E-118 | 2.231 | 3.925 | — |
| CA187951 | SPS; all-trans-nonaprenyl-diphosphate synthase [EC:2.5.1.84 2.5.1.85] | Oryza sativa | 2.0E-88 | 0.426 | — | — |

Table S1 (Continued)

| Access GenBank | Description | Blast species | e_value | D3_FC | D7_FC | D9_FC |
|---|---|---|---|---|---|---|
| CA131272 | ADT; arogenate/prephenate dehydratase [EC:4.2.1.91 4.2.1.51] | Setaria italica | 5.0E-89 | — | 0.258 | — |
| CA086723 | CYP51; sterol 14-demethylase [EC:1.14.13.70] | Zea mays | 6.0E-170 | — | 2.331 | 2.100 |
| CA219084 | ANR; anthocyanidin reductase [EC:1.3.1.77] | Setaria italica | 9.0E-162 | — | — | 0.409 |
| CA159555 | CYP90A1; cytochrome P450, family 90, subfamily A, polypeptide 1 [EC:1.14.-.-] | Zea mays | 6.0E-30 | — | 2.050 | — |
| CA230914 | NCED; 9-cis-epoxycarotenoid dioxygenase [EC:1.13.11.51] | Oryza sativa | 5.0E-49 | — | 4.625 | — |
| CA125943 | MFP2; enoyl-CoA hydratase/3-hydroxyacyl-CoA dehydrogenase [EC:4.2.1.17 1.1.1.35 1.1.1.211] | Zea mays | 0.0E+00 | — | — | 2.748 |
| CA160735 | DHDDS; ditrans,polycis-polyprenyl diphosphate synthase [EC:2.5.1.87] | Zea mays | 6.0E-110 | 2.995 | 3.423 | — |
| CA278798 | CYP79A2; phenylalanine N-monooxygenase [EC:1.14.13.124] | Zea mays | 3.0E-44 | 20.136 | — | 25.123 |
| CA238981 | thrA; bifunctional aspartokinase / homoserine dehydrogenase 1 [EC:2.7.2.4 1.1.1.3] | Zea mays | 2.0E-126 | — | — | 0.178 |
| BU103703 | ALDH18A1; delta-1-pyrroline-5-carboxylate synthetase [EC:2.7.2.11 1.2.1.41] | Sorghum bicolor | 0.0E+00 | — | 28.971 | 27.907 |
| CA286120 | PAO; pheophorbide a oxygenase [EC:1.14.12.20] | Setaria italica | 6.0E-166 | 0.229 | 0.334 | — |
| CA182293 | CYP75A; flavonoid 3',5'-hydroxylase [EC:1.14.13.88] | Zea mays | 5.0E-52 | — | — | 0.222 |
| CA174923 | LCLAT1; lysocardiolipin and lysophospholipid acyltransferase [EC:2.3.1.- 2.3.1.51] | Zea mays | 0.0E+00 | — | — | 0.484 |
| CA088276 | CAO; chlorophyllide a oxygenase [EC:1.14.13.122] | Setaria italica | 2.0E-92 | 0.428 | — | — |
| CA122302 | GOT1; aspartate aminotransferase, cytoplasmic [EC:2.6.1.1] | Setaria italica | 8.0E-136 | — | 0.395 | — |
| CA141799 | (+)-neomenthol dehydrogenase [EC:1.1.1.208] | Setaria italica | 5.0E-83 | — | 0.054 | 0.020 |
| CA133495 | KCS; 3-ketoacyl-CoA synthase [EC:2.3.1.199] | Zea mays | 5.0E-174 | — | 2.050 | — |
| CA289286 | CER1; aldehyde decarbonylase [EC:4.1.99.5] | Aegilops tauschii | 1.0E-161 | — | 15.266 | 7.528 |
| CA237557 | gpmI; 2,3-bisphosphoglycerate-independent phosphoglycerate mutase [EC:5.4.2.12] | Zea mays | 2.0E-151 | — | 0.478 | 0.437 |
| CA152408 | ADH1; alcohol dehydrogenase class-P [EC:1.1.1.1] | Arundo donax | 2.0E-112 | 0.461 | — | — |
| **Carbon metabolism** | | | | | | |
| CA206172 | MDH2; malate dehydrogenase [EC:1.1.1.37] | Zea mays | 0.0E+00 | — | — | 3.576 |
| CA246361 | malate dehydrogenase (decarboxylating) [EC:1.1.1.39] | Zea mays | 9.0E-106 | — | — | 0.486 |
| CA226469 | frmA; S-(hydroxymethyl)glutathione dehydrogenase / alcohol dehydrogenase [EC:1.1.1.284 1.1.1.1] | Zea mays | 6.0E-119 | — | 0.389 | — |
| CA086914 | GLUD1_2; glutamate dehydrogenase (NAD(P)+) [EC:1.4.1.3] | Setaria italica | 2.0E-159 | — | — | 2.059 |
| CA120928 | DLD; dihydrolipoamide dehydrogenase [EC:1.8.1.4] | Zea mays | 0.0E+00 | — | — | 2.573 |
| CA280616 | k acetyl-CoA C-acetyltransferase [EC:2.3.1.9] | Zea mays | 1.0E-161 | — | — | 3.742 |

Table S1 (Continued)

| Access GenBank | Description | Blast species | e_value | D3_FC | D7_FC | D9_FC |
|---|---|---|---|---|---|---|
| CA103835 | DLAT; pyruvate dehydrogenase E2 component (dihydrolipoamide acetyltransferase) [EC:2.3.1.12] | Zea mays | 0.0E+00 | — | — | 3.916 |
| CA094588 | cysE; serine O-acetyltransferase [EC:2.3.1.30] | Setaria italica | 2.0E-97 | — | 3.230 | 3.343 |
| CA161632 | GPT; alanine transaminase [EC:2.6.1.2] | Zea mays | 2.0E-60 | 2.979 | 3.527 | 3.138 |
| CA214701 | pfkA; 6-phosphofructokinase 1 [EC:2.7.1.11] | Zea mays | 3.0E-65 | — | — | 0.285 |
| CA282232 | PRK; phosphoribulokinase [EC:2.7.1.19] | Setaria italica | 2.0E-149 | — | — | 0.330 |
| CA189611 | PK; pyruvate kinase [EC:2.7.1.40] | Brachypodium distachyon | 2.0E-161 | — | 2.385 | 3.140 |
| CA206652 | PGK; phosphoglycerate kinase [EC:2.7.2.3] | Setaria italica | 3.0E-107 | — | 3.624 | — |
| CA191099 | ppdK; pyruvate, orthophosphate dikinase [EC:2.7.9.1] | Setaria italica | 5.0E-104 | — | — | 0.323 |
| CA224931 | PGLS; 6-phosphogluconolactonase [EC:3.1.1.31] | Zea mays | 1.0E-92 | 2.186 | 2.791 | — |
| CA111792 | formamidase [EC:3.5.1.49] | Setaria italica | 8.0E-118 | — | 0.211 | 0.104 |
| CA196624 | ppc; phosphoenolpyruvate carboxylase [EC:4.1.1.31] | Zea mays | 2.0E-138 | — | 2.469 | 2.694 |
| CA238858 | phosphoenolpyruvate carboxykinase (ATP) [EC:4.1.1.49] | Thyridolepis mitchelliana | 7.0E-164 | — | 0.218 | 0.145 |
| CA264504 | ACO; aconitate hydratase [EC:4.2.1.3] | Pinus pinaster | 4.0E-151 | — | 6.909 | 5.462 |
| CA134028 | cysK; cysteine synthase A [EC:2.5.1.47] | Zea mays | 2.0E-113 | — | 0.259 | 0.194 |
| CA134928 | rpiA; ribose 5-phosphate isomerase A [EC:5.3.1.6] | Zea mays | 3.0E-86 | 0.479 | — | — |
| CA165609 | GPI; glucose-6-phosphate isomerase [EC:5.3.1.9] | Zea mays | 9.0E-154 | — | 0.257 | 0.189 |
| CA219130 | GAPA; glyceraldehyde-3-phosphate dehydrogenase (NADP+) (phosphorylating) [EC:1.2.1.13] | Setaria italica | 1.0E-120 | — | 0.493 | 0.456 |
| CA122302 | GOT1; aspartate aminotransferase, cytoplasmic [EC:2.6.1.1] | Setaria italica | 8.0E-136 | — | 0.395 | — |
| CA237557 | gpmI; 2,3-bisphosphoglycerate-independent phosphoglycerate mutase [EC:5.4.2.12] | Zea mays | 2.0E-151 | — | 0.478 | 0.437 |
| **Ribosome** | | | | | | |
| CA158252 | RP-L1; large subunit ribosomal protein L1 | Zea mays | 9.0E-143 | — | 2.113 | — |
| CA128754 | RP-L10; large subunit ribosomal protein L10 | Zea mays | 8.0E-106 | 4.250 | 5.024 | 5.149 |
| CA128670 | RP-L11e; large subunit ribosomal protein L11e | Zea mays | 3.0E-95 | — | 0.451 | 0.201 |
| CA150444 | RP-L13; large subunit ribosomal protein L13 | Zea mays | 4.0E-138 | — | 0.376 | — |
| CA184575 | RP-L13e; large subunit ribosomal protein L13e | Zea mays | 0.0E+00 | — | — | 2.389 |
| CA074920 | RP-L16; large subunit ribosomal protein L16 | Saccharum officinarum | 1.0E-91 | — | 0.236 | 0.256 |
| CA271355 | RP-L17e; large subunit ribosomal protein L17e | Zea mays | 3.0E-86 | — | 0.237 | 0.316 |

Table S1 (Continued)

| Access GenBank | Description | Blast species | e_value | D3_FC | D7_FC | D9_FC |
|---|---|---|---|---|---|---|
| CA256509 | RP-L20; large subunit ribosomal protein L20 | Zea mays | 2.0E-73 | — | 0.473 | — |
| CA117384 | RP-L23Ae; large subunit ribosomal protein L23Ae | Zea mays | 4.0E-47 | — | 0.284 | 0.214 |
| CA127254 | RP-L23e; large subunit ribosomal protein L23e | Zea mays | 0.0E+00 | — | — | 0.406 |
| CA113414 | RP-L28e; large subunit ribosomal protein L28e | Zea mays | 4.0E-82 | — | 0.238 | 0.298 |
| CA175993 | RP-L3; large subunit ribosomal protein L3 | Zea mays | 1.0E-70 | — | 0.471 | — |
| CA289100 | RP-L35Ae; large subunit ribosomal protein L35Ae | Zea mays | 7.0E-73 | — | 0.440 | — |
| CA231357 | RP-L5e; large subunit ribosomal protein L5e | Zea mays | 0.0E+00 | — | — | 2.410 |
| CA112387 | RP-L6e; large subunit ribosomal protein L6e | Zea mays | 0.0E+00 | — | — | 0.414 |
| CA265482 | RP-L8e; large subunit ribosomal protein L8e | Setaria italica | 1.0E-167 | — | — | 0.434 |
| CA081279 | RP-L9e; large subunit ribosomal protein L9e | Zea mays | 8.0E-87 | — | 0.473 | — |
| CA168764 | RP-S12; small subunit ribosomal protein S12 | Zea mays | 4.0E-80 | — | 0.132 | — |
| CA125950 | RP-S14e; small subunit ribosomal protein S14e | Zea mays | 1.0E-79 | — | 0.373 | 0.316 |
| CA269260 | RP-S23e; small subunit ribosomal protein S23e | Zea mays | 1.0E-59 | — | 0.331 | 0.175 |
| CA149322 | RP-S27Ae; small subunit ribosomal protein S27Ae | Zea mays | 0.0E+00 | — | — | 2.201 |
| BQ533013 | RP-S3; small subunit ribosomal protein S3 | Zea mays | 4.0E-113 | — | 0.436 | — |
| CA185045 | RP-S3Ae; small subunit ribosomal protein S3Ae | Zea mays | 0.0E+00 | — | — | 0.359 |
| CA093786 | RP-S5; small subunit ribosomal protein S5 | Zea mays | 1.0E-155 | — | 0.409 | — |
| CA280092 | RP-S7e; small subunit ribosomal protein S7e | Zea mays | 7.0E-121 | — | 0.278 | 0.329 |
| CA175097 | RP-S8; small subunit ribosomal protein S8 | Setaria italica | 9.0E-157 | — | — | 0.445 |
| CA234563 | RP-S8e; small subunit ribosomal protein S8e | Zea mays | 8.0E-116 | — | 0.248 | 0.241 |
| **Phenylpropanoid biosynthesis** | | | | | | |
| CA110802 | cinnamyl-alcohol dehydrogenase [EC:1.1.1.195] | Zea mays | 7.0E-115 | — | 36.961 | 40.257 |
| CA121038 | cinnamyl-alcohol dehydrogenase [EC:1.1.1.195] | Zea mays | 2.0E-103 | — | 0.442 | — |
| CA143081 | cinnamyl-alcohol dehydrogenase [EC:1.1.1.195] | Setaria italica | 7.0E-85 | — | — | 0.375 |
| CA146051 | peroxidase [EC:1.11.1.7] | Zea mays | 4.0E-131 | 0.334 | — | |
| CA205715 | peroxidase [EC:1.11.1.7] | Setaria italica | 9.0E-99 | — | 5.305 | 3.047 |
| CA175216 | peroxidase [EC:1.11.1.7] | Sorghum bicolor | 0.0E+00 | — | — | 2.718 |

Table S1 (Continued)

| Access GenBank | Description | Blast species | e_value | D3_FC | D7_FC | D9_FC |
|---|---|---|---|---|---|---|
| CA279133 | peroxidase [EC:1.11.1.7] | Zea mays | 0.0E+00 | — | — | 0.487 |
| CA184143 | peroxidase [EC:1.11.1.7] | Zea mays | 9.0E-134 | — | 0.390 | 0.352 |
| CA273769 | peroxidase [EC:1.11.1.7] | Setaria italica | 6.0E-88 | — | 0.204 | 0.201 |
| CA274665 | peroxidase [EC:1.11.1.7] | Zea mays | 4.0E-131 | 0.334 | — | 0.364 |
| CA103087 | beta-glucosidase [EC:3.2.1.21] | Setaria italica | 8.0E-160 | — | — | 0.052 |
| CA284699 | beta-glucosidase [EC:3.2.1.21] | Oryza sativa | 9.0E-132 | 0.266 | 0.355 | 0.327 |
| CA134312 | bglB; beta-glucosidase [EC:3.2.1.21] | Zea mays | 7.0E-139 | 3.956 | 2.782 | 5.845 |
| CA217026 | bglB; beta-glucosidase [EC:3.2.1.21] | Setaria italica | 9.0E-118 | 2.231 | 3.925 | — |
| **Biosynthesis of amino acids** | | | | | | |
| CA094588 | cysE; serine O-acetyltransferase [EC:2.3.1.30] | Setaria italica | 2.0E-97 | — | 3.230 | 3.343 |
| CA184092 | metK; S-adenosylmethionine synthetase [EC:2.5.1.6] | Zea mays | 1.0E-114 | — | — | 0.067 |
| CA161632 | GPT; alanine transaminase [EC:2.6.1.2] | Zea mays | 2.0E-60 | 2.979 | 3.527 | 3.138 |
| CA228575 | TAT; tyrosine aminotransferase [EC:2.6.1.5] | Setaria italica | 0.0E+00 | — | — | 2.547 |
| CA214701 | pfkA; 6-phosphofructokinase 1 [EC:2.7.1.11] | Zea mays | 3.0E-65 | — | — | 0.285 |
| CA251534 | PK; pyruvate kinase [EC:2.7.1.40] | Zea mays | 9.0E-89 | 3.466 | — | — |
| CA228227 | PK; pyruvate kinase [EC:2.7.1.40] | Zea mays | 1.0E-110 | 2.158 | 3.444 | 2.180 |
| CA189611 | PK; pyruvate kinase [EC:2.7.1.40] | Brachypodium distachyon | 2.0E-161 | — | 2.385 | 3.140 |
| CA206652 | PGK; phosphoglycerate kinase [EC:2.7.2.3] | Setaria italica | 3.0E-107 | — | 3.624 | — |
| CA219909 | lysC; aspartate kinase [EC:2.7.2.4] | Zea mays | 8.0E-122 | 2.210 | 2.839 | 3.104 |
| CA228285 | acetolactate synthase I/II/III large subunit [EC:2.2.1.6] | Zea mays | 1.0E-88 | 2.143 | 2.261 | 2.375 |
| CA234827 | acetolactate synthase I/III small subunit [EC:2.2.1.6] | Zea mays | 2.0E-135 | — | 0.264 | 0.138 |
| CA264504 | ACO; aconitate hydratase [EC:4.2.1.3] | Pinus pinaster | 4.0E-151 | — | 6.909 | 5.462 |
| CA134028 | cysK; cysteine synthase A [EC:2.5.1.47] | Zea mays | 2.0E-113 | — | 0.259 | 0.194 |
| CA224791 | metC; cystathionine beta-lyase [EC:4.4.1.8] | Setaria italica | 0.0E+00 | — | — | 0.450 |
| CA285944 | rpiA; ribose 5-phosphate isomerase A [EC:5.3.1.6] | Zea mays | 5.0E-91 | — | 0.366 | — |
| CA134928 | rpiA; ribose 5-phosphate isomerase A [EC:5.3.1.6] | Zea mays | 3.0E-86 | 0.479 | — | — |
| CA131272 | ADT; arogenate/prephenate dehydratase [EC:4.2.1.91 4.2.1.51] | Setaria italica | 5.0E-89 | — | 0.258 | — |

Table S1 (Continued)

| Access GenBank | Description | Blast species | e_value | D3_FC | D7_FC | D9_FC |
|---|---|---|---|---|---|---|
| CA238981 | thrA; bifunctional aspartokinase / homoserine dehydrogenase 1 [EC:2.7.2.4 1.1.1.3] | Zea mays | 2.0E-126 | — | — | 0.178 |
| BU103703 | ALDH18A1; delta-1-pyrroline-5-carboxylate synthetase [EC:2.7.2.11 1.2.1.41] | Sorghum bicolor | 0.0E+00 | — | 28.971 | 27.907 |
| CA122302 | GOT1; aspartate aminotransferase, cytoplasmic [EC:2.6.1.1] | Setaria italica | 8.0E-136 | — | 0.395 | — |
| CA237557 | gpmI; 2,3-bisphosphoglycerate-independent phosphoglycerate mutase [EC:5.4.2.12] | Zea mays | 2.0E-151 | — | 0.478 | 0.437 |
| **Plant hormone signal transduction** | | | | | | |
| CA133645 | COI-1; coronatine-insensitive protein 1 | Zea mays | 0.0E+00 | — | — | 2.077 |
| CA075535 | JAZ; jasmonate ZIM domain-containing protein | Zea mays | 4.0E-39 | — | 0.346 | 0.296 |
| CA285332 | BSK; BR-signaling kinase [EC:2.7.11.1] | Setaria italica | 2.0E-164 | — | 2.167 | 2.071 |
| CA252857 | IAA; auxin-responsive protein IAA | Setaria italica | 1.0E-24 | — | 0.208 | 0.225 |
| CA162286 | GH3; auxin responsive GH3 gene family | Zea mays | 9.0E-136 | — | 2.103 | — |
| CA095141 | ABF; ABA responsive element binding factor | Zea mays | 0.0E+00 | — | — | 2.199 |
| CA129160 | SNRK2; serine/threonine-protein kinase SRK2 [EC:2.7.11.1] | Setaria italica | 1.0E-162 | — | 2.182 | — |
| CA280103 | SNRK2; serine/threonine-protein kinase SRK2 [EC:2.7.11.1] | Saccharum officinarum | 0.0E+00 | — | 4.093 | 2.443 |
| CA196644 | PP2C; protein phosphatase 2C [EC:3.1.3.16] | Setaria italica | 0.0E+00 | — | — | 2.052 |
| CA246148 | PP2C; protein phosphatase 2C [EC:3.1.3.16] | Zea mays | 3.0E-59 | — | 0.470 | — |
| CA078060 | PP2C; protein phosphatase 2C [EC:3.1.3.16] | Zea mays | 1.0E-86 | 16.298 | 14.231 | 10.549 |
| CA093454 | protein phosphatase 2C (PP2C) | Zea mays | 7.0E-47 | 10.262 | 9.540 | 52.923 |
| CA113863 | cytokinin response regulator2 (crr2) | Zea mays | 7.0E-118 | — | — | 2.270 |
| CA154411 | two-component response regulator ARR9-like | Setaria italica | 9.0E-66 | — | — | 0.084 |
| CA191067 | ARR-A; two-component response regulator ARR-A family | Zea mays | 7.0E-93 | — | 0.127 | 0.145 |
| CA238851 | RR1 - Corn type-A response regulator | Zea mays | 2.0E-54 | — | — | 0.107 |
| **Phenylalanine metabolism** | | | | | | |
| CA192696 | AOC3; primary-amine oxidase [EC:1.4.3.21] | Setaria italica | 6.0E-114 | — | — | 2.050 |
| CA146051 | peroxidase [EC:1.11.1.7] | Zea mays | 4.0E-131 | 0.334 | — | |
| CA205715 | peroxidase [EC:1.11.1.7] | Setaria italica | 9.0E-99 | — | 5.305 | 3.047 |
| CA279133 | peroxidase [EC:1.11.1.7] | Zea mays | 0.0E+00 | — | — | 0.487 |
| CA184143 | peroxidase [EC:1.11.1.7] | Zea mays | 9.0E-134 | — | 0.390 | 0.352 |

Table S1 (Continued)

| Access GenBank | Description | Blast species | e_value | D3_FC | D7_FC | D9_FC |
|---|---|---|---|---|---|---|
| CA274665 | peroxidase [EC:1.11.1.7] | Zea mays | 4.0E-131 | 0.334 | — | 0.364 |
| CA175216 | peroxidase [EC:1.11.1.7] | Sorghum bicolor | 0.0E+00 | — | — | 2.718 |
| CA273769 | peroxidase [EC:1.11.1.7] | Setaria italica | 6.0E-88 | — | 0.204 | 0.201 |
| CA228575 | TAT; tyrosine aminotransferase [EC:2.6.1.5] | Setaria italica | 0.0E+00 | — | — | 2.547 |
| CA122302 | GOT1; aspartate aminotransferase, cytoplasmic [EC:2.6.1.1] | Setaria italica | 8.0E-136 | — | 0.395 | — |
| **Glycolysis / Gluconeogenesis** | | | | | | |
| CA226469 | frmA; S-(hydroxymethyl)glutathione dehydrogenase / alcohol dehydrogenase [EC:1.1.1.284 1.1.1.1] | Zea mays | 6.0E-119 | — | 0.389 | — |
| CA120928 | DLD; dihydrolipoamide dehydrogenase [EC:1.8.1.4] | Zea mays | 0.0E+00 | — | — | 2.573 |
| CA103835 | DLAT; pyruvate dehydrogenase E2 component (dihydrolipoamide acetyltransferase) [EC:2.3.1.12] | Zea mays | 0.0E+00 | — | — | 3.916 |
| CA214701 | pfkA; 6-phosphofructokinase 1 [EC:2.7.1.11] | Zea mays | 3.0E-65 | — | — | 0.285 |
| CA189611 | PK; pyruvate kinase [EC:2.7.1.40] | Brachypodium distachyon | 2.0E-161 | — | 2.385 | 3.140 |
| CA206652 | PGK; phosphoglycerate kinase [EC:2.7.2.3] | Setaria italica | 3.0E-107 | — | 3.624 | — |
| CA238858 | phosphoenolpyruvate carboxykinase (ATP) [EC:4.1.1.49] | Thyridolepis mitchelliana | 7.0E-164 | — | 0.218 | 0.145 |
| CA261190 | galM; aldose 1-epimerase [EC:5.1.3.3] | | | 0.415 | — | — |
| CA289954 | glucose-6-phosphate 1-epimerase [EC:5.1.3.15] | Setaria italica | 7.0E-134 | — | 0.269 | 0.302 |
| CA272115 | GPI; glucose-6-phosphate isomerase [EC:5.3.1.9] | Zea mays | 6.0E-131 | — | 0.215 | 0.296 |
| CA165609 | GPI; glucose-6-phosphate isomerase [EC:5.3.1.9] | Zea mays | 9.0E-154 | — | 0.257 | 0.189 |
| CA251914 | MINPP1; multiple inositol-polyphosphate phosphatase / 2,3-bisphosphoglycerate 3-phosphatase [EC:3.1.3.62 3.1.3.80] | Setaria italica | 2.0E-83 | — | 2.154 | — |
| CA237557 | gpmI; 2,3-bisphosphoglycerate-independent phosphoglycerate mutase [EC:5.4.2.12] | Zea mays | 2.0E-151 | — | 0.478 | 0.437 |
| CA152408 | ADH1; alcohol dehydrogenase class-P [EC:1.1.1.1] | Arundo donax | 2.0E-112 | 0.461 | — | — |
| **Pyruvate metabolism** | | | | | | |
| CA206172 | MDH2; malate dehydrogenase [EC:1.1.1.37] | Zea mays | 0.0E+00 | — | — | 3.576 |
| CA246361 | malate dehydrogenase (decarboxylating) [EC:1.1.1.39] | Zea mays | 9.0E-106 | — | — | 0.486 |
| CA120928 | DLD; dihydrolipoamide dehydrogenase [EC:1.8.1.4] | Zea mays | 0.0E+00 | — | — | 2.573 |
| CA280616 | acetyl-CoA C-acetyltransferase [EC:2.3.1.9] | Zea mays | 1.0E-161 | — | — | 3.742 |
| CA103835 | DLAT; pyruvate dehydrogenase E2 component (dihydrolipoamide acetyltransferase) [EC:2.3.1.12] | Zea mays | 0.0E+00 | — | — | 3.916 |

Table S1 (Continued)

| Access GenBank | Description | Blast species | e_value | D3_FC | D7_FC | D9_FC |
|---|---|---|---|---|---|---|
| CA251534 | PK; pyruvate kinase [EC:2.7.1.40] | Zea mays | 9.0E-89 | 3.466 | — | — |
| CA228227 | PK; pyruvate kinase [EC:2.7.1.40] | Zea mays | 1.0E-110 | 2.158 | 3.444 | 2.180 |
| CA189611 | PK; pyruvate kinase [EC:2.7.1.40] | Brachypodium distachyon | 2.0E-161 | — | 2.385 | 3.140 |
| CA191099 | ppdK; pyruvate, orthophosphate dikinase [EC:2.7.9.1] | Setaria italica | 5.0E-104 | — | — | 0.323 |
| CA196624 | ppc; phosphoenolpyruvate carboxylase [EC:4.1.1.31] | Zea mays | 2.0E-138 | — | 2.469 | 2.694 |
| CA238858 | phosphoenolpyruvate carboxykinase (ATP) [EC:4.1.1.49] | Thyridolepis mitchelliana | 7.0E-164 | — | 0.218 | 0.145 |
| **Starch and sucrose metabolism** | | | | | | |
| CA175853 | sucrose synthase [EC:2.4.1.13] | Zea mays | 7.0E-104 | — | 0.354 | — |
| CA196779 | sucrose synthase [EC:2.4.1.13] | Setaria italica | 1.0E-101 | 2.410 | 3.846 | 4.111 |
| CA258249 | starch synthase [EC:2.4.1.21] | Zea mays | 5.0E-36 | 0.483 | 0.429 | 0.474 |
| BQ533079 | beta-amylase [EC:3.2.1.2] | Zea mays | 1.0E-132 | — | 2.381 | — |
| CA103087 | beta-glucosidase [EC:3.2.1.21] | Setaria italica | 8.0E-160 | — | — | 0.052 |
| CA284699 | beta-glucosidase [EC:3.2.1.21] | Oryza sativa | 9.0E-132 | 0.266 | 0.355 | 0.327 |
| CA165609 | GPI; glucose-6-phosphate isomerase [EC:5.3.1.9] | Zea mays | 9.0E-154 | — | 0.257 | 0.189 |
| CA272115 | GPI; glucose-6-phosphate isomerase [EC:5.3.1.9] | Zea mays | 6.0E-131 | — | 0.215 | 0.296 |
| CA134312 | bglB; beta-glucosidase [EC:3.2.1.21] | Zea mays | 7.0E-139 | 3.956 | 2.782 | 5.845 |
| CA217026 | bglB; beta-glucosidase [EC:3.2.1.21] | Setaria italica | 9.0E-118 | 2.231 | 3.925 | — |
| CA254458 | UXS1; UDP-glucuronate decarboxylase [EC:4.1.1.35] | Zea mays | 0.0E+00 | — | — | 2.225 |
| CA295036 | UXS1; UDP-glucuronate decarboxylase [EC:4.1.1.35] | Oryza sativa | 3.0E-151 | — | 0.303 | — |
| CA177178 | XYL4; beta-D-xylosidase 4 [EC:3.2.1.37] | Setaria italica | 0.0E+00 | — | — | 2.581 |
| CA293142 | TPS; trehalose 6-phosphate synthase/phosphatase [EC:2.4.1.15 3.1.3.12] | Zea mays | 4.0E-164 | 0.071 | — | 0.132 |
| **Cysteine and methionine metabolism** | | | | | | |
| CA206172 | MDH2; malate dehydrogenase [EC:1.1.1.37] | Zea mays | 0.0E+00 | — | — | 3.576 |
| CA094588 | cysE; serine O-acetyltransferase [EC:2.3.1.30] | Setaria italica | 2.0E-97 | — | 3.230 | 3.343 |
| CA184092 | metK; S-adenosylmethionine synthetase [EC:2.5.1.6] | Zea mays | 1.0E-114 | — | — | 0.067 |
| CA228575 | TAT; tyrosine aminotransferase [EC:2.6.1.5] | Setaria italica | 0.0E+00 | — | — | 2.547 |
| CA219909 | lysC; aspartate kinase [EC:2.7.2.4] | Zea mays | 8.0E-122 | 2.210 | 2.839 | 3.104 |

Table S1 (Continued)

| Access GenBank | Description | Blast species | e_value | D3_FC | D7_FC | D9_FC |
|---|---|---|---|---|---|---|
| CA134028 | cysK; cysteine synthase A [EC:2.5.1.47] | Zea mays | 2.0E-113 | — | 0.259 | 0.194 |
| CA224791 | metC; cystathionine beta-lyase [EC:4.4.1.8] | Setaria italica | 0.0E+00 | — | — | 0.450 |
| CA164646 | mtnD; 1,2-dihydroxy-3-keto-5-methylthiopentene dioxygenase [EC:1.13.11.53 1.13.11.54] | Zea mays | 1.0E-99 | — | 0.437 | 0.316 |
| CA238981 | thrA; bifunctional aspartokinase / homoserine dehydrogenase 1 [EC:2.7.2.4 1.1.1.3] | Zea mays | 2.0E-126 | — | — | 0.178 |
| CA122302 | GOT1; aspartate aminotransferase, cytoplasmic [EC:2.6.1.1] | Setaria italica | 8.0E-136 | — | 0.395 | — |
| **Oxidative phosphorylation** | | | | | | |
| CA291830 | ppa; inorganic pyrophosphatase [EC:3.6.1.1] | Zea mays | 9.0E-153 | — | 0.370 | — |
| BU103680 | ppa; inorganic pyrophosphatase [EC:3.6.1.1] | Setaria italica | 0.0E+00 | — | — | 3.210 |
| CA234649 | H+-transporting ATPase [EC:3.6.3.6] | Setaria italica | 0.0E+00 | — | — | 0.447 |
| CA285765 | ATPF1G; F-type H+-transporting ATPase subunit gamma | Zea mays | 4.0E-110 | — | 0.198 | 0.202 |
| CA301517 | ATPeV1B; V-type H+-transporting ATPase subunit B | Arabidopsis thaliana | 1.0E-133 | — | 0.314 | 0.219 |
| CA183028 | ATPeV0C; V-type H+-transporting ATPase 16kDa proteolipid subunit | Setaria italica | 0.0E+00 | — | — | 0.477 |
| CA236146 | NDUFA6; NADH dehydrogenase (ubiquinone) 1 alpha subcomplex subunit 6 | Setaria italica | 9.0E-90 | — | 0.187 | 0.188 |
| CA126813 | NDUFB7; NADH dehydrogenase (ubiquinone) 1 beta subcomplex subunit 7 | Sorghum bicolor | 3.0E-77 | — | — | 0.121 |
| CA227722 | NDUFB10; NADH dehydrogenase (ubiquinone) 1 beta subcomplex subunit 10 | Zea mays | 0.0E+00 | — | — | 0.442 |
| **Purine metabolism** | | | | | | |
| CA206289 | purH; phosphoribosylaminoimidazolecarboxamide formyltransferase / IMP cyclohydrolase [EC:2.1.2.3 3.5.4.10] | Saccharum hybrid cultivar | 3.1E-155 | 2.199 | — | — |
| CA182186 | cysC; adenylylsulfate kinase [EC:2.7.1.25] | Setaria italica | 3.0E-67 | — | 5.454 | — |
| CA251534 | PK; pyruvate kinase [EC:2.7.1.40] | Zea mays | 9.0E-89 | 3.466 | — | — |
| CA228227 | PK; pyruvate kinase [EC:2.7.1.40] | Zea mays | 1.0E-110 | 2.158 | 3.444 | 2.180 |
| CA189611 | PK; pyruvate kinase [EC:2.7.1.40] | Brachypodium distachyon | 2.0E-161 | — | 2.385 | 3.140 |
| CA124145 | ndk; nucleoside-diphosphate kinase [EC:2.7.4.6] | Zea mays | 2.0E-142 | — | — | 0.399 |
| CA243497 | purM; phosphoribosylformylglycinamidine cyclo-ligase [EC:6.3.3.1] | Setaria italica | 8.0E-81 | — | 0.383 | 0.375 |
| CA174659 | guaA; GMP synthase (glutamine-hydrolysing) [EC:6.3.5.2] | Setaria italica | 9.0E-65 | — | 0.346 | 0.380 |
| CA221482 | purL; phosphoribosylformylglycinamidine synthase [EC:6.3.5.3] | Oryza sativa | 7.0E-132 | — | 0.497 | — |
| CA267750 | POLD1; DNA polymerase delta subunit 1 [EC:2.7.7.7] | Zea mays | 0.0E+00 | — | — | 2.178 |

Table S1 (Continued)

| Access GenBank | Description | Blast species | e_value | D3_FC | D7_FC | D9_FC |
|---|---|---|---|---|---|---|
| CA148996 | RPB11; DNA-directed RNA polymerase II subunit RPB11 | Setaria italica | 2.0E-129 | — | — | 2.108 |
| CA111337 | RPB9; DNA-directed RNA polymerase II subunit RPB9 | Zea mays | 4.0E-157 | — | — | 0.439 |
| CA238403 | rpoA; DNA-directed RNA polymerase subunit alpha [EC:2.7.7.6] | Saccharum officinarum | 1.0E-139 | — | 0.400 | — |
| CA236105 | rpoC; DNA-directed RNA polymerase subunit beta' [EC:2.7.7.6] | Zea mays | 2.0E-126 | — | 0.366 | — |
| CA093575 | PAPSS; 3'-phosphoadenosine 5'-phosphosulfate synthase [EC:2.7.7.4 2.7.1.25] | Zea mays | 1.0E-74 | — | 0.195 | 0.119 |
| **Carbon fixation in photosynthetic organisms** | | | | | | |
| CA206172 | MDH2; malate dehydrogenase [EC:1.1.1.37] | Zea mays | 0.0E+00 | — | — | 3.576 |
| CA246361 | malate dehydrogenase (decarboxylating) [EC:1.1.1.39] | Zea mays | 9.0E-106 | — | — | 0.486 |
| CA161632 | GPT; alanine transaminase [EC:2.6.1.2] | Zea mays | 2.0E-60 | 2.979 | 3.527 | 3.138 |
| CA282232 | PRK; phosphoribulokinase [EC:2.7.1.19] | Setaria italica | 2.0E-149 | — | — | 0.330 |
| CA206652 | PGK; phosphoglycerate kinase [EC:2.7.2.3] | Setaria italica | 3.0E-107 | — | 3.624 | — |
| CA191099 | ppdK; pyruvate, orthophosphate dikinase [EC:2.7.9.1] | Setaria italica | 5.0E-104 | — | — | 0.323 |
| CA196624 | ppc; phosphoenolpyruvate carboxylase [EC:4.1.1.31] | Zea mays | 2.0E-138 | — | 2.469 | 2.694 |
| CA238858 | phosphoenolpyruvate carboxykinase (ATP) [EC:4.1.1.49] | Thyridolepis mitchelliana | 7.0E-164 | — | 0.218 | 0.145 |
| CA219130 | GAPA; glyceraldehyde-3-phosphate dehydrogenase (NADP+) (phosphorylating) [EC:1.2.1.13] | Setaria italica | 1.0E-120 | — | 0.493 | 0.456 |
| CA122302 | GOT1; aspartate aminotransferase, cytoplasmic [EC:2.6.1.1] | Setaria italica | 8.0E-136 | — | 0.395 | — |
| CA134928 | rpiA; ribose 5-phosphate isomerase A [EC:5.3.1.6] | Zea mays | 3.0E-86 | 0.479 | — | — |
| CA285944 | rpiA; ribose 5-phosphate isomerase A [EC:5.3.1.6] | Zea mays | 5.0E-91 | — | 0.366 | — |
| **Protein processing in endoplasmic reticulum** | | | | | | |
| CA280099 | SKP1; S-phase kinase-associated protein 1 | Setaria italica | 4.0E-89 | — | 0.437 | 0.417 |
| CA275126 | HSPA1_8; heat shock 70kDa protein 1/8 | Saussurea medusa | 9.0E-134 | — | 4.025 | 3.059 |
| CA125208 | HSPA1_8; heat shock 70kDa protein 1/8 | Zea mays | 3.0E-127 | — | 0.448 | — |
| CA238353 | htpG; molecular chaperone HtpG | Setaria italica | 0.0E+00 | — | — | 2.018 |
| CA110498 | UBE2D_E; ubiquitin-conjugating enzyme E2 D/E [EC:6.3.2.19] | Zea mays | 2.0E-100 | — | 0.331 | 0.300 |
| CA206309 | SAR1; GTP-binding protein SAR1 [EC:3.6.5.-] | | | — | — | 4.436 |
| CA081124 | SSR1; translocon-associated protein subunit alpha | Zea mays | 2.0E-101 | — | 0.486 | 0.285 |
| CA261170 | HSP20; HSP20 family protein | Saccharum officinarum | 2.0E-78 | — | 6.360 | — |

Table S1 (Continued)

| Access GenBank | Description | Blast species | e_value | D3_FC | D7_FC | D9_FC |
|---|---|---|---|---|---|---|
| CA239113 | SEC23; protein transport protein SEC23 | Setaria italica | 2.0E-113 | — | 2.061 | 2.176 |
| **Spliceosome** | | | | | | |
| CA125208 | HSPA1_8; heat shock 70kDa protein 1/8 | Zea mays | 3.0E-127 | — | 0.448 | — |
| CA275126 | HSPA1_8; heat shock 70kDa protein 1/8 | Saussurea medusa | 9.0E-134 | — | 4.025 | 3.059 |
| CA194528 | LSM2; U6 snRNA-associated Sm-like protein LSm2 | Setaria italica | 2.0E-113 | — | — | 0.400 |
| CA088031 | HNRNPA1_3; heterogeneous nuclear ribonucleoprotein A1/A3 | Zea mays | 9.0E-78 | — | 0.405 | 0.307 |
| CA124076 | SMNDC1; survival of motor neuron-related-splicing factor 30 | Zea mays | 3.0E-97 | — | — | 0.450 |
| CA173531 | RBM17; splicing factor 45 | Zea mays | 1.0E-48 | — | 2.038 | — |
| CA298822 | SYF1; pre-mRNA-splicing factor SYF1 | Setaria italica | 2.0E-105 | — | 0.496 | — |
| CA177160 | ACIN1; apoptotic chromatin condensation inducer in the nucleus | Zea mays | 6.0E-102 | — | 2.685 | 2.256 |
| CA226172 | SFRS2; splicing factor, arginine/serine-rich 2 | Setaria italica | 0.0E+00 | — | — | 0.392 |
| CA148625 | SFRS7; splicing factor, arginine/serine-rich 7 | Setaria italica | 1.0E-32 | — | 0.254 | 0.409 |
| **Amino sugar and nucleotide sugar metabolism** | | | | | | |
| CA222079 | UAP1; UDP-N-acetylglucosamine/UDP-N-acetylgalactosamine diphosphorylase [EC:2.7.7.23 2.7.7.83] | Zea mays | 7.0E-154 | — | 0.422 | 0.367 |
| CA134439 | chitinase [EC:3.2.1.14] | Setaria italica | 0.0E+00 | — | — | 2.402 |
| CA098256 | galE; UDP-glucose 4-epimerase [EC:5.1.3.2] | Zea mays | 6.0E-132 | 6.496 | 6.528 | — |
| CA272115 | GPI; glucose-6-phosphate isomerase [EC:5.3.1.9] | Zea mays | 6.0E-131 | — | 0.215 | 0.296 |
| CA165609 | GPI; glucose-6-phosphate isomerase [EC:5.3.1.9] | Zea mays | 9.0E-154 | — | 0.257 | 0.189 |
| CA121841 | L-arabinokinase [EC:2.7.1.46] | Setaria italica | 2.0E-140 | 2.577 | — | — |
| CA177178 | XYL4; beta-D-xylosidase 4 [EC:3.2.1.37] | Setaria italica | 0.0E+00 | — | — | 2.581 |
| **Fatty acid metabolism** | | | | | | |
| CA261850 | acyl-CoA oxidase [EC:1.3.3.6] | Zea mays | 3.0E-97 | — | 0.250 | 0.224 |
| CA280616 | acetyl-CoA C-acetyltransferase [EC:2.3.1.9] | Zea mays | 1.0E-161 | — | — | 3.742 |
| CA262389 | ACSL; long-chain acyl-CoA synthetase [EC:6.2.1.3] | Oryza sativa | 1.0E-137 | — | 2.908 | 3.587 |
| CA219064 | ACSL; long-chain acyl-CoA synthetase [EC:6.2.1.3] | Zea mays | 2.0E-74 | 3.420 | — | — |
| CA205998 | ACSL; long-chain acyl-CoA synthetase [EC:6.2.1.3] | Setaria italica | 0.0E+00 | — | — | 3.059 |
| CA266796 | FAD8; omega-3 fatty acid desaturase (delta-15 desaturase) [EC:1.14.19.-] | Zea mays | 1.0E-40 | — | — | 0.267 |

Table S1 (Continued)

| Access GenBank | Description | Blast species | e_value | D3_FC | D7_FC | D9_FC |
|---|---|---|---|---|---|---|
| CA125943 | MFP2; enoyl-CoA hydratase/3-hydroxyacyl-CoA dehydrogenase [EC:4.2.1.17 1.1.1.35 1.1.1.211] | Zea mays | 0.0E+00 | — | — | 2.748 |
| **Flavonoid biosynthesis** | | | | | | |
| CA219084 | ANR; anthocyanidin reductase [EC:1.3.1.77] | Setaria italica | 9.0E-162 | — | — | 0.409 |
| CA219145 | ANR; anthocyanidin reductase [EC:1.3.1.77] | Setaria italica | 6.0E-100 | — | 3.329 | 3.791 |
| **Glutathione metabolism** | | | | | | |
| CA089083 | GSR; glutathione reductase (NADPH) [EC:1.8.1.7] | Zea mays | 2.0E-29 | — | — | 0.213 |
| CA133463 | GSR; glutathione reductase (NADPH) [EC:1.8.1.7] | Setaria italica | 0.0E+00 | — | — | 0.498 |
| CA146699 | GST; glutathione S-transferase [EC:2.5.1.18] | Zea mays | 5.0E-69 | — | 0.372 | 0.325 |
| CA131351 | GST; glutathione S-transferase [EC:2.5.1.18] | Zea mays | 2.0E-84 | — | 0.258 | — |
| CA130806 | GST; glutathione S-transferase [EC:2.5.1.18] | Zea mays | 1.0E-144 | — | — | 2.755 |
| CA179421 | CARP; leucyl aminopeptidase [EC:3.4.11.1] | Setaria italica | 1.0E-130 | — | 2.087 | — |
| **Glycine, serine and threonine metabolism** | | | | | | |
| CA192696 | AOC3; primary-amine oxidase [EC:1.4.3.21] | Setaria italica | 6.0E-114 | — | — | 2.050 |
| CA120928 | DLD; dihydrolipoamide dehydrogenase [EC:1.8.1.4] | Zea mays | 0.0E+00 | — | — | 2.573 |
| CA096192 | AGXT2; alanine-glyoxylate transaminase / (R)-3-amino-2-methylpropionate-pyruvate transaminase [EC:2.6.1.44 2.6.1.40] | Zea mays | 1.0E-73 | 6.685 | 7.437 | 4.073 |
| CA219909 | lysC; aspartate kinase [EC:2.7.2.4] | Zea mays | 8.0E-122 | 2.210 | 2.839 | 3.104 |
| CA238981 | thrA; bifunctional aspartokinase / homoserine dehydrogenase 1 [EC:2.7.2.4 1.1.1.3] | Zea mays | 2.0E-126 | — | — | 0.178 |
| CA237557 | gpmI; 2,3-bisphosphoglycerate-independent phosphoglycerate mutase [EC:5.4.2.12] | Zea mays | 2.0E-151 | — | 0.478 | 0.437 |
| **2-Oxocarboxylic acid metabolism** | | | | | | |
| CA161632 | GPT; alanine transaminase [EC:2.6.1.2] | Zea mays | 2.0E-60 | 2.979 | 3.527 | 3.138 |
| CA219909 | lysC; aspartate kinase [EC:2.7.2.4] | Zea mays | 8.0E-122 | 2.210 | 2.839 | 3.104 |
| CA228285 | acetolactate synthase I/II/III large subunit [EC:2.2.1.6] | Zea mays | 1.0E-88 | 2.143 | 2.261 | 2.375 |
| CA234827 | ko:K01653 E2.2.1.6S; acetolactate synthase I/III small subunit [EC:2.2.1.6] | Zea mays | 2.0E-135 | — | 0.264 | 0.138 |
| CA264504 | ACO; aconitate hydratase [EC:4.2.1.3] | Pinus pinaster | 4.0E-151 | — | 6.909 | 5.462 |
| CA122302 | GOT1; aspartate aminotransferase, cytoplasmic [EC:2.6.1.1] | Setaria italica | 8.0E-136 | — | 0.395 | — |

Table S1 (Continued)

| Access GenBank | Description | Blast species | e_value | D3_FC | D7_FC | D9_FC |
|---|---|---|---|---|---|---|
| **Cyanoamino acid metabolism** | | | | | | |
| CA103087 | beta-glucosidase [EC:3.2.1.21] | Setaria italica | 8.0E-160 | — | — | 0.052 |
| CA284699 | beta-glucosidase [EC:3.2.1.21] | Oryza sativa | 9.0E-132 | 0.266 | 0.355 | 0.327 |
| CA111792 | formamidase [EC:3.5.1.49] | Setaria italica | 8.0E-118 | — | 0.211 | 0.104 |
| CA134312 | bglB; beta-glucosidase [EC:3.2.1.21] | Zea mays | 7.0E-139 | 3.956 | 2.782 | 5.845 |
| CA217026 | bglB; beta-glucosidase [EC:3.2.1.21] | Setaria italica | 9.0E-118 | 2.231 | 3.925 | — |
| **Fatty acid degradation** | | | | | | |
| CA226469 | frmA; S-(hydroxymethyl)glutathione dehydrogenase / alcohol dehydrogenase [EC:1.1.1.284 1.1.1.1] | Zea mays | 6.0E-119 | — | 0.389 | — |
| CA261850 | acyl-CoA oxidase [EC:1.3.3.6] | Zea mays | 3.0E-97 | — | 0.250 | 0.224 |
| CA280616 | acetyl-CoA C-acetyltransferase [EC:2.3.1.9] | Zea mays | 1.0E-161 | — | — | 3.742 |
| CA205998 | ACSL; long-chain acyl-CoA synthetase [EC:6.2.1.3] | Setaria italica | 0.0E+00 | — | — | 3.059 |
| CA219064 | ACSL; long-chain acyl-CoA synthetase [EC:6.2.1.3] | Zea mays | 2.0E-74 | 3.420 | — | — |
| CA262389 | ACSL; long-chain acyl-CoA synthetase [EC:6.2.1.3] | Oryza sativa | 1.0E-137 | — | 2.908 | 3.587 |
| CA125943 | MFP2; enoyl-CoA hydratase/3-hydroxyacyl-CoA dehydrogenase [EC:4.2.1.17 1.1.1.35 1.1.1.211] | Zea mays | 0.0E+00 | — | — | 2.748 |
| CA152408 | ADH1; alcohol dehydrogenase class-P [EC:1.1.1.1] | Arundo donax | 2.0E-112 | 0.461 | — | — |
| **Plant-pathogen interaction** | | | | | | |
| CA124384 | CALM; calmodulin | Zea mays | 0.0E+00 | — | — | 2.105 |
| CA238353 | htpG; molecular chaperone HtpG | Setaria italica | 0.0E+00 | — | — | 2.018 |
| CA269216 | CNGF; cyclic nucleotide gated channel, other eukaryote | Hordeum vulgare | 5.0E-142 | 0.433 | 0.487 | 0.467 |
| CA135934 | CPK; calcium-dependent protein kinase [EC:2.7.11.1] | Zea mays | 0.0E+00 | — | — | 2.348 |
| CA157947 | CERK1; chitin elicitor receptor kinase 1 | Zea mays | 0.0E+00 | — | — | 0.431 |
| CA103645 | PTI1; pto-interacting protein 1 [EC:2.7.11.1] | Zea mays | 4.0E-104 | 5.573 | 3.755 | — |
| **Ubiquitin mediated proteolysis** | | | | | | |
| CA280099 | SKP1; S-phase kinase-associated protein 1 | Setaria italica | 4.0E-89 | — | 0.437 | 0.417 |
| CA264410 | UBE1; ubiquitin-activating enzyme E1 [EC:6.3.2.19] | Zea mays | 0.0E+00 | — | — | 0.465 |
| CA078404 | SIAH1; E3 ubiquitin-protein ligase SIAH1 [EC:6.3.2.19] | Setaria italica | 5.0E-148 | — | — | 0.267 |
| CA090272 | SIAH1; E3 ubiquitin-protein ligase SIAH1 [EC:6.3.2.19] | Setaria italica | 1.0E-116 | — | — | 0.461 |

Table S1 (Continued)

| Access GenBank | Description | Blast species | e_value | D3_FC | D7_FC | D9_FC |
|---|---|---|---|---|---|---|
| CA110498 | UBE2D_E; ubiquitin-conjugating enzyme E2 D/E [EC:6.3.2.19] | Zea mays | 2.0E-100 | — | 0.331 | 0.300 |
| CA269860 | TRIP12; E3 ubiquitin-protein ligase TRIP12 [EC:6.3.2.19] | Setaria italica | 2.0E-132 | — | 0.498 | — |
| **Alanine, aspartate and glutamate metabolism** | | | | | | |
| CA086914 | GLUD1_2; glutamate dehydrogenase (NAD(P)+) [EC:1.4.1.3] | Setaria italica | 2.0E-159 | — | — | 2.059 |
| CA161632 | GPT; alanine transaminase [EC:2.6.1.2] | Zea mays | 2.0E-60 | 2.979 | 3.527 | 3.138 |
| CA096192 | AGXT2; alanine-glyoxylate transaminase / (R)-3-amino-2-methylpropionate-pyruvate transaminase [EC:2.6.1.44 2.6.1.40] | Zea mays | 1.0E-73 | 6.685 | 7.437 | 4.073 |
| CA134623 | asnB; asparagine synthase (glutamine-hydrolysing) [EC:6.3.5.4] | Zea mays | 3.0E-141 | — | 10.266 | — |
| CA265748 | asnB; asparagine synthase (glutamine-hydrolysing) [EC:6.3.5.4] | Zea mays | 3.0E-125 | — | 0.353 | 0.223 |
| CA122302 | GOT1; aspartate aminotransferase, cytoplasmic [EC:2.6.1.1] | Setaria italica | 8.0E-136 | — | 0.395 | — |
| **Circadian rhythm - plant** | | | | | | |
| CA216109 | PHYB; phytochrome B | Sorghum bicolor | 2.0E-137 | — | 0.234 | 0.113 |
| CA065738 | TOC1; pseudo-response regulator 1 | Setaria italica | 4.0E-35 | — | 0.422 | 0.386 |
| CA284643 | FT; protein FLOWERING LOCUS T | Zea mays | 2.0E-98 | 0.270 | 0.268 | 0.346 |
| **Citrate cycle (TCA cycle)** | | | | | | |
| CA206172 | MDH2; malate dehydrogenase [EC:1.1.1.37] | Zea mays | 0.0E+00 | — | — | 3.576 |
| CA120928 | DLD; dihydrolipoamide dehydrogenase [EC:1.8.1.4] | Zea mays | 0.0E+00 | — | — | 2.573 |
| CA103835 | DLAT; pyruvate dehydrogenase E2 component (dihydrolipoamide acetyltransferase) [EC:2.3.1.12] | Zea mays | 0.0E+00 | — | — | 3.916 |
| CA238858 | phosphoenolpyruvate carboxykinase (ATP) [EC:4.1.1.49] | Thyridolepis mitchelliana | 7.0E-164 | — | 0.218 | 0.145 |
| CA264504 | ACO; aconitate hydratase [EC:4.2.1.3] | Pinus pinaster | 4.0E-151 | — | 6.909 | 5.462 |
| **mRNA surveillance pathway** | | | | | | |
| CA167585 | SMG1; PI-3-kinase-related kinase SMG-1 | Setaria italica | 3.0E-138 | — | 0.297 | 0.428 |
| CA128904 | PPP2R5; serine/threonine-protein phosphatase 2A regulatory subunit B' | Setaria italica | 0.0E+00 | — | — | 3.183 |
| CA177160 | ACIN1; apoptotic chromatin condensation inducer in the nucleus | Zea mays | 6.0E-102 | — | 2.685 | 2.256 |
| CA239950 | MSI; RNA-binding protein Musashi | Setaria italica | 0.0E+00 | — | — | 2.022 |
| CA206273 | PFS2; polyadenylation factor subunit 2 | Zea mays | 0.0E+00 | — | — | 2.230 |
| **Peroxisome** | | | | | | |
| CA261850 | acyl-CoA oxidase [EC:1.3.3.6] | Zea mays | 3.0E-97 | — | 0.250 | 0.224 |

Table S1 (Continued)

| Access GenBank | Description | Blast species | e_value | D3_FC | D7_FC | D9_FC |
|---|---|---|---|---|---|---|
| CA205998 | ACSL; long-chain acyl-CoA synthetase [EC:6.2.1.3] | Setaria italica | 0.0E+00 | — | — | 3.059 |
| CA219064 | ACSL; long-chain acyl-CoA synthetase [EC:6.2.1.3] | Zea mays | 2.0E-74 | 3.420 | — | — |
| CA262389 | ACSL; long-chain acyl-CoA synthetase [EC:6.2.1.3] | Oryza sativa | 1.0E-137 | — | 2.908 | 3.587 |
| CA140681 | MPV17; protein Mpv17 | Zea mays | 3.0E-99 | — | — | 0.166 |
| **Proteasome** | | | | | | |
| CA293413 | PSMB1; 20S proteasome subunit beta 6 [EC:3.4.25.1] | Zea mays | 0.0E+00 | — | — | 0.409 |
| CA123048 | PSMD14; 26S proteasome regulatory subunit N11 | Zea mays | 0.0E+00 | — | — | 2.369 |
| CA252377 | PSMD8; 26S proteasome regulatory subunit N12 | Zea mays | 3.0E-147 | — | 0.374 | — |
| CA284586 | PSMD1; 26S proteasome regulatory subunit N2 | Setaria italica | 2.0E-92 | — | 0.413 | — |
| CA164209 | PSMD11; 26S proteasome regulatory subunit N6 | Setaria italica | 0.0E+00 | — | — | 0.400 |
| CA086721 | PSMD6; 26S proteasome regulatory subunit N7 | Zea mays | 8.0E-159 | — | 0.322 | 0.333 |
| CA280753 | PSMC1; 26S proteasome regulatory subunit T2 | Zea mays | 0.0E+00 | — | — | 2.188 |
| **RNA degradation** | | | | | | |
| CA214701 | pfkA; 6-phosphofructokinase 1 [EC:2.7.1.11] | Zea mays | 3.0E-65 | — | — | 0.285 |
| CA244080 | CNOT7_8; CCR4-NOT transcription complex subunit 7/8 | Zea mays | 4.0E-155 | — | — | 0.411 |
| CA120984 | MPHOSPH6; M-phase phosphoprotein 6, animal type | Sorghum bicolor | 5.0E-86 | — | 0.460 | 0.287 |
| CA194528 | LSM2; U6 snRNA-associated Sm-like protein LSm2 | Setaria italica | 2.0E-113 | — | — | 0.400 |
| CA266953 | DHX36; ATP-dependent RNA helicase DHX36 [EC:3.6.4.13] | Zea mays | 0.0E+00 | — | — | 0.493 |
| **Arginine and proline metabolism** | | | | | | |
| CA134936 | prolyl 4-hydroxylase [EC:1.14.11.2] | Zea mays | 0.0E+00 | — | — | 2.261 |
| CA079225 | SMOX; spermine oxidase [EC:1.5.3.16 1.5.3.-] | Setaria italica | 1.0E-169 | — | — | 3.262 |
| BU103703 | ALDH18A1; delta-1-pyrroline-5-carboxylate synthetase [EC:2.7.2.11 1.2.1.41] | Sorghum bicolor | 0.0E+00 | — | 28.971 | 27.907 |
| CA122302 | GOT1; aspartate aminotransferase, cytoplasmic [EC:2.6.1.1] | Setaria italica | 8.0E-136 | — | 0.395 | — |
| **Endocytosis** | | | | | | |
| CA073821 | PIP5K; 1-phosphatidylinositol-4-phosphate 5-kinase [EC:2.7.1.68] | Zea mays | 8.0E-100 | — | 4.302 | — |
| CA125208 | HSPA1_8; heat shock 70kDa protein 1/8 | Zea mays | 3.0E-127 | — | 0.448 | — |
| CA275126 | HSPA1_8; heat shock 70kDa protein 1/8 | Saussurea medusa | 9.0E-134 | — | 4.025 | 3.059 |

Table S1 (Continued)

| Access GenBank | Description | Blast species | e_value | D3_FC | D7_FC | D9_FC |
|---|---|---|---|---|---|---|
| CA203005 | ARF1; ADP-ribosylation factor 1 | Zea mays | 3.0E-66 | — | — | 0.318 |
| CA083172 | SNF8; ESCRT-II complex subunit VPS22 | Sorghum bicolor | 1.0E-92 | — | 0.418 | 0.216 |
| CA282978 | ARFGAP2_3; ADP-ribosylation factor GTPase-activating protein 2/3 | Zea mays | 3.0E-72 | 2.357 | — | — |
| **Glyoxylate and dicarboxylate metabolism** | | | | | | |
| CA206172 | MDH2; malate dehydrogenase [EC:1.1.1.37] | Zea mays | 0.0E+00 | — | — | 3.576 |
| CA120928 | DLD; dihydrolipoamide dehydrogenase [EC:1.8.1.4] | Zea mays | 0.0E+00 | — | — | 2.573 |
| CA280616 | acetyl-CoA C-acetyltransferase [EC:2.3.1.9] | Zea mays | 1.0E-161 | — | — | 3.742 |
| CA111792 | formamidase [EC:3.5.1.49] | Setaria italica | 8.0E-118 | — | 0.211 | 0.104 |
| CA264504 | ACO; aconitate hydratase [EC:4.2.1.3] | Pinus pinaster | 4.0E-151 | — | 6.909 | 5.462 |
| **Other glycan degradation** | | | | | | |
| CA276171 | lacZ; beta-galactosidase [EC:3.2.1.23] | Setaria italica | 2.0E-154 | — | — | 0.255 |
| CA158042 | alpha-mannosidase [EC:3.2.1.24] | Triticum urartu | 1.0E-112 | 3.309 | 5.442 | 3.345 |
| CA242862 | GLB1; beta-galactosidase [EC:3.2.1.23] | Zea mays | 0.0E+00 | — | — | 0.490 |
| **Phagosome** | | | | | | |
| CA280162 | PIKFYVE; 1-phosphatidylinositol-3-phosphate 5-kinase [EC:2.7.1.150] | Zea mays | 0.0E+00 | — | — | 2.476 |
| CA301517 | ATPeV1B; V-type H+-transporting ATPase subunit B | Arabidopsis thaliana | 1.0E-133 | — | 0.314 | 0.219 |
| CA183028 | ATPeV0C; V-type H+-transporting ATPase 16kDa proteolipid subunit | Setaria italica | 0.0E+00 | — | — | 0.477 |
| CA279490 | RAC1; Ras-related C3 botulinum toxin substrate 1 | Zea mays | 1.0E-38 | 0.319 | 0.398 | 0.111 |
| **Pyrimidine metabolism** | | | | | | |
| CA245326 | udk; uridine kinase [EC:2.7.1.48] | Zea mays | 9.0E-78 | 2.511 | — | — |
| CA124145 | ndk; nucleoside-diphosphate kinase [EC:2.7.4.6] | Zea mays | 2.0E-142 | — | — | 0.399 |
| CA267750 | POLD1; DNA polymerase delta subunit 1 [EC:2.7.7.7] | Zea mays | 0.0E+00 | — | — | 2.178 |
| CA148996 | RPB11; DNA-directed RNA polymerase II subunit RPB11 | Setaria italica | 2.0E-129 | — | — | 2.108 |
| CA111337 | RPB9; DNA-directed RNA polymerase II subunit RPB9 | Zea mays | 4.0E-157 | — | — | 0.439 |
| CA238403 | rpoA; DNA-directed RNA polymerase subunit alpha [EC:2.7.7.6] | Saccharum officinarum | 1.0E-139 | — | 0.400 | — |
| CA236105 | rpoC; DNA-directed RNA polymerase subunit beta' [EC:2.7.7.6] | Zea mays | 2.0E-126 | — | 0.366 | — |

Table S1 (Continued)

| Access GenBank | Description | Blast species | e_value | D3_FC | D7_FC | D9_FC |
|---|---|---|---|---|---|---|
| **Selenocompound metabolism** | | | | | | |
| CA224791 | metC; cystathionine beta-lyase [EC:4.4.1.8] | Setaria italica | 0.0E+00 | — | — | 0.450 |
| CA093180 | MARS; methionyl-tRNA synthetase [EC:6.1.1.10] | Zea mays | 0.0E+00 | — | — | 0.487 |
| CA242837 | methionine S-methyltransferase [EC:2.1.1.12] | Zea mays | 4.0E-115 | — | 0.385 | — |
| CA093575 | PAPSS; 3'-phosphoadenosine 5'-phosphosulfate synthase [EC:2.7.7.4 2.7.1.25] | Zea mays | 1.0E-74 | — | 0.195 | 0.119 |
| **Aminoacyl-tRNA biosynthesis** | | | | | | |
| CA093180 | MARS; methionyl-tRNA synthetase [EC:6.1.1.10] | Zea mays | 0.0E+00 | — | — | 0.487 |
| CA117569 | SARS; seryl-tRNA synthetase [EC:6.1.1.11] | Zea mays | 4.0E-87 | — | — | 0.263 |
| CA214590 | GARS; glycyl-tRNA synthetase [EC:6.1.1.14] | Setaria italica | 4.0E-137 | — | 0.469 | 0.439 |
| CA151646 | EARS; glutamyl-tRNA synthetase [EC:6.1.1.17] | Setaria italica | 7.0E-148 | — | 0.245 | — |
| CA179697 | KARS; lysyl-tRNA synthetase, class II [EC:6.1.1.6] | Triticum urartu | 2.0E-44 | — | 0.496 | — |
| **ABC transporters** | | | | | | |
| CA080185 | ABCB1; ATP-binding cassette, subfamily B (MDR/TAP), member 1 [EC:3.6.3.44] | Zea mays | 3.0E-55 | — | 0.460 | — |
| CA151192 | ABCB1; ATP-binding cassette, subfamily B (MDR/TAP), member 1 [EC:3.6.3.44] | Sorghum bicolor | 5.0E-73 | — | 0.393 | 0.305 |
| **alpha-Linolenic acid metabolism** | | | | | | |
| CA261850 | acyl-CoA oxidase [EC:1.3.3.6] | Zea mays | 3.0E-97 | — | 0.250 | 0.224 |
| CA125943 | MFP2; enoyl-CoA hydratase/3-hydroxyacyl-CoA dehydrogenase [EC:4.2.1.17 1.1.1.35 1.1.1.211] | Zea mays | 0.0E+00 | — | — | 2.748 |
| CA152408 | ADH1; alcohol dehydrogenase class-P [EC:1.1.1.1] | Arundo donax | 2.0E-112 | 0.461 | — | — |
| **Ascorbate and aldarate metabolism** | | | | | | |
| CA182682 | GalDH; L-galactose dehydrogenase [EC:1.1.1.316] | Sorghum bicolor | 0.0E+00 | — | — | 5.896 |
| **Base excision repair** | | | | | | |
| CA267750 | POLD1; DNA polymerase delta subunit 1 [EC:2.7.7.7] | Zea mays | 0.0E+00 | — | — | 2.178 |
| **beta-Alanine metabolism** | | | | | | |
| CA079225 | SMOX; spermine oxidase [EC:1.5.3.16 1.5.3.-] | Setaria italica | 1.0E-169 | — | — | 3.262 |
| CA192696 | AOC3; primary-amine oxidase [EC:1.4.3.21] | Setaria italica | 6.0E-114 | — | — | 2.050 |
| **Biosynthesis of unsaturated fatty acids** | | | | | | |
| CA261850 | acyl-CoA oxidase [EC:1.3.3.6] | Zea mays | 3.0E-97 | — | 0.250 | 0.224 |

Table S1 (Continued)

| Access GenBank | Description | Blast species | e_value | D3_FC | D7_FC | D9_FC |
|---|---|---|---|---|---|---|
| CA266796 | FAD8; omega-3 fatty acid desaturase (delta-15 desaturase) [EC:1.14.19.-] | Zea mays | 1.0E-40 | — | — | 0.267 |
| CA278038 | FAD8; omega-3 fatty acid desaturase (delta-15 desaturase) [EC:1.14.19.-] | Zea mays | 6.0E-34 | — | 3.540 | — |
| **Biotin metabolism** | | | | | | |
| CA237512 | bioF; 8-amino-7-oxononanoate synthase [EC:2.3.1.47] | Zea mays | 3.0E-84 | — | — | 0.205 |
| **Brassinosteroid biosynthesis** | | | | | | |
| CA159555 | CYP90A1; cytochrome P450, family 90, subfamily A, polypeptide 1 [EC:1.14.-.-] | Zea mays | 6.0E-30 | — | 2.050 | — |
| **Butanoate metabolism** | | | | | | |
| CA280616 | acetyl-CoA C-acetyltransferase [EC:2.3.1.9] | Zea mays | 1.0E-161 | — | — | 3.742 |
| CA228285 | acetolactate synthase I/II/III large subunit [EC:2.2.1.6] | Zea mays | 1.0E-88 | 2.143 | 2.261 | 2.375 |
| CA234827 | acetolactate synthase I/III small subunit [EC:2.2.1.6] | Zea mays | 2.0E-135 | — | 0.264 | 0.138 |
| **C5-Branched dibasic acid metabolism** | | | | | | |
| CA228285 | acetolactate synthase I/II/III large subunit [EC:2.2.1.6] | Zea mays | 1.0E-88 | 2.143 | 2.261 | 2.375 |
| CA234827 | acetolactate synthase I/III small subunit [EC:2.2.1.6] | Zea mays | 2.0E-135 | — | 0.264 | 0.138 |
| **Carotenoid biosynthesis** | | | | | | |
| CA230914 | NCED; 9-cis-epoxycarotenoid dioxygenase [EC:1.13.11.51] | Oryza sativa | 5.0E-49 | — | 4.625 | — |
| CA122105 | (+)-abscisic acid 8'-hydroxylase [EC:1.14.13.93] | Zea mays | 2.0E-117 | — | 5.092 | — |
| CA174832 | CCD8; carlactone synthase / all-trans-10'-apo-beta-carotenal 13,14-cleaving dioxygenase [EC:1.13.11.69 1.13.11.70] | Setaria italica | 4.0E-47 | — | 9.828 | — |
| **Cutin, suberine and wax biosynthesis** | | | | | | |
| CA289286 | CER1; aldehyde decarbonylase [EC:4.1.99.5] | Aegilops tauschii | 1.0E-161 | — | 15.266 | 7.528 |
| **Degradation of aromatic compounds** | | | | | | |
| CA226469 | frmA; S-(hydroxymethyl)glutathione dehydrogenase / alcohol dehydrogenase [EC:1.1.1.284 1.1.1.1] | Zea mays | 6.0E-119 | — | 0.389 | — |
| **Diterpenoid biosynthesis** | | | | | | |
| CA223665 | ent-copalyl diphosphate synthase [EC:5.5.1.13] | Zea mays | 5.0E-149 | — | 0.058 | 0.032 |
| CA186391 | GA3; ent-kaurene oxidase [EC:1.14.13.78] | Zea mays | 3.0E-35 | — | 2.035 | — |
| **DNA replication** | | | | | | |
| CA267750 | POLD1; DNA polymerase delta subunit 1 [EC:2.7.7.7] | Zea mays | 0.0E+00 | — | — | 2.178 |
| CA119570 | RFA1; replication factor A1 | Zea mays | 0.0E+00 | — | — | 0.360 |

Table S1 (Continued)

| Access GenBank | Description | Blast species | e_value | D3_FC | D7_FC | D9_FC |
|---|---|---|---|---|---|---|
| **Fatty acid biosynthesis** | | | | | | |
| CA205998 | ACSL; long-chain acyl-CoA synthetase [EC:6.2.1.3] | Setaria italica | 0.0E+00 | — | — | 3.059 |
| CA219064 | ACSL; long-chain acyl-CoA synthetase [EC:6.2.1.3] | Zea mays | 2.0E-74 | 3.420 | — | — |
| CA262389 | ACSL; long-chain acyl-CoA synthetase [EC:6.2.1.3] | Oryza sativa | 1.0E-137 | — | 2.908 | 3.587 |
| **Fatty acid elongation** | | | | | | |
| CA133495 | KCS; 3-ketoacyl-CoA synthase [EC:2.3.1.199] | Zea mays | 5.0E-174 | — | 2.050 | — |
| **Fructose and mannose metabolism** | | | | | | |
| CA134332 | SORD; L-iditol 2-dehydrogenase [EC:1.1.1.14] | Zea mays | 0.0E+00 | — | — | 0.431 |
| CA214701 | pfkA; 6-phosphofructokinase 1 [EC:2.7.1.11] | Zea mays | 3.0E-65 | — | — | 0.285 |
| CA099192 | kopyrophosphate--fructose-6-phosphate 1-phosphotransferase [EC:2.7.1.90] | Zea mays | 5.0E-114 | — | — | 0.214 |
| **Galactose metabolism** | | | | | | |
| CA214701 | pfkA; 6-phosphofructokinase 1 [EC:2.7.1.11] | Zea mays | 3.0E-65 | — | — | 0.285 |
| CA276171 | lacZ; beta-galactosidase [EC:3.2.1.23] | Setaria italica | 2.0E-154 | — | — | 0.255 |
| CA098256 | galE; UDP-glucose 4-epimerase [EC:5.1.3.2] | Zea mays | 6.0E-132 | 6.496 | 6.528 | — |
| CA261190 | galM; aldose 1-epimerase [EC:5.1.3.3] | | | 0.415 | — | — |
| CA117454 | alpha-galactosidase [EC:3.2.1.22] | Brachypodium distachyon | 4.0E-87 | 4.650 | 4.845 | — |
| CA285497 | alpha-galactosidase [EC:3.2.1.22] | Zea mays | 6.0E-104 | 0.262 | 0.235 | — |
| CA242862 | GLB1; beta-galactosidase [EC:3.2.1.23] | Zea mays | 0.0E+00 | — | — | 0.490 |
| **Glycerolipid metabolism** | | | | | | |
| CA233761 | phospholipid:diacylglycerol acyltransferase [EC:2.3.1.158] | Setaria italica | 7.0E-101 | — | 0.329 | 0.252 |
| CA117454 | alpha-galactosidase [EC:3.2.1.22] | Brachypodium distachyon | 4.0E-87 | 4.650 | 4.845 | — |
| CA285497 | alpha-galactosidase [EC:3.2.1.22] | Zea mays | 6.0E-104 | 0.262 | 0.235 | — |
| CA264490 | digalactosyldiacylglycerol synthase [EC:2.4.1.241] | Setaria italica | 1.0E-110 | — | — | 0.342 |
| CA174923 | LCLAT1; lysocardiolipin and lysophospholipid acyltransferase [EC:2.3.1.- 2.3.1.51] | Zea mays | 0.0E+00 | — | — | 0.484 |
| **Glycerophospholipid metabolism** | | | | | | |
| CA174923 | LCLAT1; lysocardiolipin and lysophospholipid acyltransferase [EC:2.3.1.- 2.3.1.51] | Zea mays | 0.0E+00 | — | — | 0.484 |
| **Glycosaminoglycan degradation** | | | | | | |
| CA242862 | GLB1; beta-galactosidase [EC:3.2.1.23] | Zea mays | 0.0E+00 | — | — | 0.490 |

Table S1 (Continued)

| Access GenBank | Description | Blast species | e_value | D3_FC | D7_FC | D9_FC |
|---|---|---|---|---|---|---|
| **Glycosphingolipid biosynthesis - ganglio series** | | | | | | |
| CA242862 | GLB1; beta-galactosidase [EC:3.2.1.23] | Zea mays | 0.0E+00 | — | — | 0.490 |
| **Glycosphingolipid biosynthesis - globo series** | | | | | | |
| CA285497 | alpha-galactosidase [EC:3.2.1.22] | Zea mays | 6.0E-104 | 0.262 | 0.235 | — |
| CA117454 | alpha-galactosidase [EC:3.2.1.22] | Brachypodium distachyon | 4.0E-87 | 4.650 | 4.845 | — |
| **Glycosylphosphatidylinositol(GPI)-anchor biosynthesis** | | | | | | |
| CA094332 | DPM2; dolichyl-phosphate mannosyltransferase polypeptide 2, regulatory subunit | Setaria italica | 1.0E-90 | — | — | 0.360 |
| **Homologous recombination** | | | | | | |
| CA267750 | POLD1; DNA polymerase delta subunit 1 [EC:2.7.7.7] | Zea mays | 0.0E+00 | — | — | 2.178 |
| CA080115 | TOP3; DNA topoisomerase III [EC:5.99.1.2] | Setaria italica | 9.0E-151 | — | — | 2.009 |
| CA119570 | RFA1; replication factor A1 | Zea mays | 0.0E+00 | — | — | 0.360 |
| **Inositol phosphate metabolism** | | | | | | |
| CA073821 | PIP5K; 1-phosphatidylinositol-4-phosphate 5-kinase [EC:2.7.1.68] | Zea mays | 8.0E-100 | — | 4.302 | — |
| CA280162 | PIKFYVE; 1-phosphatidylinositol-3-phosphate 5-kinase [EC:2.7.1.150] | Zea mays | 0.0E+00 | — | — | 2.476 |
| CA085551 | myo-inositol-1-phosphate synthase [EC:5.5.1.4] | Zea mays | 2.0E-86 | 2.252 | — | — |
| CA251914 | MINPP1; multiple inositol-polyphosphate phosphatase / 2,3-bisphosphoglycerate 3-phosphatase [EC:3.1.3.62 3.1.3.80] | Setaria italica | 2.0E-83 | — | 2.154 | — |
| **Isoquinoline alkaloid biosynthesis** | | | | | | |
| CA192696 | AOC3; primary-amine oxidase [EC:1.4.3.21] | Setaria italica | 6.0E-114 | — | — | 2.050 |
| CA228575 | TAT; tyrosine aminotransferase [EC:2.6.1.5] | Setaria italica | 0.0E+00 | — | — | 2.547 |
| CA122302 | GOT1; aspartate aminotransferase, cytoplasmic [EC:2.6.1.1] | Setaria italica | 8.0E-136 | — | 0.395 | — |
| **Lysine biosynthesis** | | | | | | |
| CA219909 | lysC; aspartate kinase [EC:2.7.2.4] | Zea mays | 8.0E-122 | 2.210 | 2.839 | 3.104 |
| CA238981 | thrA; bifunctional aspartokinase / homoserine dehydrogenase 1 [EC:2.7.2.4 1.1.1.3] | Zea mays | 2.0E-126 | — | — | 0.178 |
| **Lysine degradation** | | | | | | |
| CA280616 | acetyl-CoA C-acetyltransferase [EC:2.3.1.9] | Zea mays | 1.0E-161 | — | — | 3.742 |
| CA199176 | EHMT; euchromatic histone-lysine N-methyltransferase [EC:2.1.1.43] | Zea mays | 5.0E-154 | — | 2.538 | — |
| CA203135 | EHMT; euchromatic histone-lysine N-methyltransferase [EC:2.1.1.43] | Setaria italica | 0.0E+00 | — | — | 0.400 |

Table S1 (Continued)

| Access GenBank | Description | Blast species | e_value | D3_FC | D7_FC | D9_FC |
|---|---|---|---|---|---|---|
| **Mismatch repair** | | | | | | |
| CA267750 | POLD1; DNA polymerase delta subunit 1 [EC:2.7.7.7] | Zea mays | 0.0E+00 | — | — | 2.178 |
| CA119570 | RFA1; replication factor A1 | Zea mays | 0.0E+00 | — | — | 0.360 |
| CA293362 | MSH2; DNA mismatch repair protein MSH2 | Setaria italica | 2.0E-58 | — | 0.388 | 0.199 |
| **N-Glycan biosynthesis** | | | | | | |
| CA094332 | DPM2; dolichyl-phosphate mannosyltransferase polypeptide 2, regulatory subunit | Setaria italica | 1.0E-90 | — | — | 0.360 |
| **Nicotinate and nicotinamide metabolism** | | | | | | |
| CA095742 | pncB; nicotinate phosphoribosyltransferase [EC:6.3.4.21] | Setaria italica | 9.0E-140 | 6.993 | 8.378 | — |
| CA158312 | nadC; nicotinate-nucleotide pyrophosphorylase (carboxylating) [EC:2.4.2.19] | Oryza sativa | 6.0E-59 | — | 0.388 | 0.372 |
| **Nitrogen metabolism** | | | | | | |
| CA086914 | GLUD1_2; glutamate dehydrogenase (NAD(P)+) [EC:1.4.1.3] | Setaria italica | 2.0E-159 | — | — | 2.059 |
| CA233400 | nirA; ferredoxin-nitrite reductase [EC:1.7.7.1] | Zea mays | 4.0E-102 | — | 0.472 | — |
| CA111792 | formamidase [EC:3.5.1.49] | Setaria italica | 8.0E-118 | — | 0.211 | 0.104 |
| CA123382 | cynT; carbonic anhydrase [EC:4.2.1.1] | Zea mays | 3.0E-39 | 4.529 | 4.472 | — |
| **Nucleotide excision repair** | | | | | | |
| CA267750 | POLD1; DNA polymerase delta subunit 1 [EC:2.7.7.7] | Zea mays | 0.0E+00 | — | — | 2.178 |
| CA119570 | RFA1; replication factor A1 | Zea mays | 0.0E+00 | — | — | 0.360 |
| **One carbon pool by folate** | | | | | | |
| CA206289 | purH; phosphoribosylaminoimidazolecarboxamide formyltransferase / IMP cyclohydrolase EC:2.1.2.3 3.5.4.10] | Saccharum hybrid cultivar | 3.1E-155 | 2.199 | — | — |
| **Pantothenate and CoA biosynthesis** | | | | | | |
| CA228285 | acetolactate synthase I/II/III large subunit [EC:2.2.1.6] | Zea mays | 1.0E-88 | 2.143 | 2.261 | 2.375 |
| CA234827 | acetolactate synthase I/III small subunit [EC:2.2.1.6] | Zea mays | 2.0E-135 | — | 0.264 | 0.138 |
| **Pentose and glucuronate interconversions** | | | | | | |
| CA134332 | SORD; L-iditol 2-dehydrogenase [EC:1.1.1.14] | Zea mays | 0.0E+00 | — | — | 0.431 |
| **Pentose phosphate pathway** | | | | | | |
| CA214701 | pfkA; 6-phosphofructokinase 1 [EC:2.7.1.11] | Zea mays | 3.0E-65 | — | — | 0.285 |
| CA224931 | PGLS; 6-phosphogluconolactonase [EC:3.1.1.31] | Zea mays | 1.0E-92 | 2.186 | 2.791 | — |

Table S1 (Continued)

| Access GenBank | Description | Blast species | e_value | D3_FC | D7_FC | D9_FC |
|---|---|---|---|---|---|---|
| CA134928 | rpiA; ribose 5-phosphate isomerase A [EC:5.3.1.6] | Zea mays | 3.0E-86 | 0.479 | — | — |
| CA285944 | rpiA; ribose 5-phosphate isomerase A [EC:5.3.1.6] | Zea mays | 5.0E-91 | — | 0.366 | — |
| CA165609 | GPI; glucose-6-phosphate isomerase [EC:5.3.1.9] | Zea mays | 9.0E-154 | — | 0.257 | 0.189 |
| CA272115 | GPI; glucose-6-phosphate isomerase [EC:5.3.1.9] | Zea mays | 6.0E-131 | — | 0.215 | 0.296 |
| **Phenylalanine, tyrosine and tryptophan biosynthesis** | | | | | | |
| CA228575 | TAT; tyrosine aminotransferase [EC:2.6.1.5] | Setaria italica | 0.0E+00 | — | — | 2.547 |
| CA131272 | ADT; arogenate/prephenate dehydratase [EC:4.2.1.91 4.2.1.51] | Setaria italica | 5.0E-89 | — | 0.258 | — |
| CA122302 | GOT1; aspartate aminotransferase, cytoplasmic [EC:2.6.1.1] | Setaria italica | 8.0E-136 | — | 0.395 | — |
| **Phosphatidylinositol signaling system** | | | | | | |
| CA073821 | PIP5K; 1-phosphatidylinositol-4-phosphate 5-kinase [EC:2.7.1.68] | Zea mays | 8.0E-100 | — | 4.302 | — |
| CA280162 | PIKFYVE; 1-phosphatidylinositol-3-phosphate 5-kinase [EC:2.7.1.150] | Zea mays | 0.0E+00 | — | — | 2.476 |
| CA124384 | CALM; calmodulin | Zea mays | 0.0E+00 | — | — | 2.105 |
| **Porphyrin and chlorophyll metabolism** | | | | | | |
| CA160929 | PPOX; oxygen-dependent protoporphyrinogen oxidase [EC:1.3.3.4] | Zea mays | 3.0E-86 | 2.979 | 2.502 | 2.267 |
| CA100020 | hemH; ferrochelatase [EC:4.99.1.1] | Sorghum bicolor | 0.0E+00 | — | — | 0.399 |
| CA151646 | EARS; glutamyl-tRNA synthetase [EC:6.1.1.17] | Setaria italica | 7.0E-148 | — | 0.245 | — |
| CA294821 | magnesium-protoporphyrin IX monomethyl ester (oxidative) cyclase [EC:1.14.13.81] | Setaria italica | 2.0E-179 | 0.369 | — | — |
| CA286120 | PAO; pheophorbide a oxygenase [EC:1.14.12.20] | Setaria italica | 6.0E-166 | 0.229 | 0.334 | — |
| CA088276 | CAO; chlorophyllide a oxygenase [EC:1.14.13.122] | Setaria italica | 2.0E-92 | 0.428 | — | — |
| **Propanoate metabolism** | | | | | | |
| CA280616 | acetyl-CoA C-acetyltransferase [EC:2.3.1.9] | Zea mays | 1.0E-161 | — | — | 3.742 |
| Protein export | | | | | | |
| CA189840 | SRP9; signal recognition particle subunit SRP9 | Zea mays | 7.0E-61 | 2.453 | 2.501 | — |
| CA226363 | SRPRB; signal recognition particle receptor subunit beta | Setaria italica | 4.0E-118 | — | — | 0.272 |
| **Regulation of autophagy** | | | | | | |
| CA152856 | PRKAA; 5'-AMP-activated protein kinase, catalytic alpha subunit [EC:2.7.11.11] | Zea mays | 5.0E-169 | — | — | 0.275 |

Table S1 (Continued)

| Access GenBank | Description | Blast species | e_value | D3_FC | D7_FC | D9_FC |
|---|---|---|---|---|---|---|
| **Ribosome biogenesis in eukaryotes** | | | | | | |
| CA067416 | UTP10; U3 small nucleolar RNA-associated protein 10 | Sorghum bicolor | 2.0E-123 | — | — | 0.223 |
| CA294502 | IMP4; U3 small nucleolar ribonucleoprotein protein IMP4 | Setaria italica | 8.0E-116 | — | 2.549 | — |
| **RNA polymerase** | | | | | | |
| CA148996 | RPB11; DNA-directed RNA polymerase II subunit RPB11 | Setaria italica | 2.0E-129 | — | — | 2.108 |
| CA111337 | RPB9; DNA-directed RNA polymerase II subunit RPB9 | Zea mays | 4.0E-157 | — | — | 0.439 |
| CA238403 | rpoA; DNA-directed RNA polymerase subunit alpha [EC:2.7.7.6] | Saccharum officinarum | 1.0E-139 | — | 0.400 | — |
| CA236105 | rpoC; DNA-directed RNA polymerase subunit beta' [EC:2.7.7.6] | Zea mays | 2.0E-126 | — | 0.366 | — |
| **RNA transport** | | | | | | |
| CA258465 | EIF4A; translation initiation factor 4A | Zea mays | 9.0E-135 | — | 0.496 | 0.413 |
| CA133372 | EIF4E; translation initiation factor 4E | Zea mays | 5.0E-116 | — | 0.396 | 0.254 |
| CA177160 | ACIN1; apoptotic chromatin condensation inducer in the nucleus | Zea mays | 6.0E-102 | — | 2.685 | 2.256 |
| **SNARE interactions in vesicular transport** | | | | | | |
| CA123082 | STX1B_2_3; syntaxin 1B/2/3 | Zea mays | 3.0E-65 | 2.747 | — | — |
| CA234603 | GOSR2; golgi SNAP receptor complex member 2 | Zea mays | 3.0E-40 | — | 0.444 | — |
| CA222131 | SYP5; syntaxin of plants SYP5 | Setaria italica | 1.0E-109 | — | 0.265 | — |
| **Sphingolipid metabolism** | | | | | | |
| CA276171 | lacZ; beta-galactosidase [EC:3.2.1.23] | Setaria italica | 2.0E-154 | — | — | 0.255 |
| CA268784 | DEGS; sphingolipid delta-4 desaturase [EC:1.14.-.-] | Setaria italica | 1.0E-149 | — | 3.884 | — |
| CA114344 | SUR2; sphinganine C4-monooxygenase [EC:1.14.13.169] | Setaria italica | 5.0E-120 | — | 2.280 | — |
| CA117454 | alpha-galactosidase [EC:3.2.1.22] | Brachypodium distachyon | 4.0E-87 | 4.650 | 4.845 | — |
| CA285497 | alpha-galactosidase [EC:3.2.1.22] | Zea mays | 6.0E-104 | 0.262 | 0.235 | — |
| CA242862 | GLB1; beta-galactosidase [EC:3.2.1.23] | Zea mays | 0.0E+00 | — | — | 0.490 |
| **Steroid biosynthesis** | | | | | | |
| CA159555 | CYP90A1; cytochrome P450, family 90, subfamily A, polypeptide 1 [EC:1.14.-.-] | Zea mays | 6.0E-30 | — | 2.050 | — |

Table S1 (Continued)

| Access GenBank | Description | Blast species | e_value | D3_FC | D7_FC | D9_FC |
|---|---|---|---|---|---|---|
| **Sulfur metabolism** | | | | | | |
| CA094588 | cysE; serine O-acetyltransferase [EC:2.3.1.30] | Setaria italica | 2.0E-97 | — | 3.230 | 3.343 |
| CA182186 | cysC; adenylylsulfate kinase [EC:2.7.1.25] | Setaria italica | 3.0E-67 | — | 5.454 | — |
| CA134028 | cysK; cysteine synthase A [EC:2.5.1.47] | Zea mays | 2.0E-113 | — | 0.259 | 0.194 |
| CA093575 | PAPSS; 3'-phosphoadenosine 5'-phosphosulfate synthase [EC:2.7.7.4 2.7.1.25] | Zea mays | 1.0E-74 | — | 0.195 | 0.119 |
| **Terpenoid backbone biosynthesis** | | | | | | |
| CA280616 | acetyl-CoA C-acetyltransferase [EC:2.3.1.9] | Zea mays | 1.0E-161 | — | — | 3.742 |
| CA187951 | SPS; all-trans-nonaprenyl-diphosphate synthase [EC:2.5.1.84 2.5.1.85] | Oryza sativa | 2.0E-88 | 0.426 | — | — |
| CA160735 | DHDDS; ditrans,polycis-polyprenyl diphosphate synthase [EC:2.5.1.87] | Zea mays | 6.0E-110 | 2.995 | 3.423 | — |
| **Tropane, piperidine and pyridine alkaloid biosynthesis** | | | | | | |
| CA192696 | AOC3; primary-amine oxidase [EC:1.4.3.21] | Setaria italica | 6.0E-114 | — | — | 2.050 |
| CA228575 | TAT; tyrosine aminotransferase [EC:2.6.1.5] | Setaria italica | 0.0E+00 | — | — | 2.547 |
| CA122302 | GOT1; aspartate aminotransferase, cytoplasmic [EC:2.6.1.1] | Setaria italica | 8.0E-136 | — | 0.395 | — |
| **Tryptophan metabolism** | | | | | | |
| CA280616 | acetyl-CoA C-acetyltransferase [EC:2.3.1.9] | Zea mays | 1.0E-161 | — | — | 3.742 |
| **Tyrosine metabolism** | | | | | | |
| CA226469 | frmA; S-(hydroxymethyl)glutathione dehydrogenase / alcohol dehydrogenase [EC:1.1.1.284 1.1.1.1] | Zea mays | 6.0E-119 | — | 0.389 | — |
| CA192696 | AOC3; primary-amine oxidase [EC:1.4.3.21] | Setaria italica | 6.0E-114 | — | — | 2.050 |
| CA228575 | TAT; tyrosine aminotransferase [EC:2.6.1.5] | Setaria italica | 0.0E+00 | — | — | 2.547 |
| CA122302 | GOT1; aspartate aminotransferase, cytoplasmic [EC:2.6.1.1] | Setaria italica | 8.0E-136 | — | 0.395 | — |
| CA152408 | ADH1; alcohol dehydrogenase class-P [EC:1.1.1.1] | Arundo donax | 2.0E-112 | 0.461 | — | — |
| **Ubiquinone and other terpenoid-quinone biosynthesis** | | | | | | |
| CA228575 | TAT; tyrosine aminotransferase [EC:2.6.1.5] | Setaria italica | 0.0E+00 | — | — | 2.547 |
| **Valine, leucine and isoleucine biosynthesis** | | | | | | |
| CA228285 | acetolactate synthase I/II/III large subunit [EC:2.2.1.6] | Zea mays | 1.0E-88 | 2.143 | 2.261 | 2.375 |
| CA234827 | acetolactate synthase I/III small subunit [EC:2.2.1.6] | Zea mays | 2.0E-135 | — | 0.264 | 0.138 |

Table S1 (Continued)

| Access GenBank | Description | Blast species | e_value | D3_FC | D7_FC | D9_FC |
|---|---|---|---|---|---|---|
| **Valine, leucine and isoleucine degradation** | | | | | | |
| CA120928 | DLD; dihydrolipoamide dehydrogenase [EC:1.8.1.4] | Zea mays | 0.0E+00 | — | — | 2.573 |
| CA280616 | acetyl-CoA C-acetyltransferase [EC:2.3.1.9] | Zea mays | 1.0E-161 | — | — | 3.742 |
| CA122991 | AUH; methylglutaconyl-CoA hydratase [EC:4.2.1.18] | Zea mays | 4.0E-87 | — | 2.418 | — |

Access GenBank: Access number of gene to NCBI database; e-value: e-value from BlastX; D3/7/9_FC: fold change of differential expressed genes in each sampled days, respectively

Table S2 Validation of selected candidate genes using real time-PCR

| Access No. of GenBank | Description | Primer | Time point | Microarray | qRT-PCR |
|---|---|---|---|---|---|
| EF189713 | Glyceraldehyde-3-phosphate dehydrogenase (GAPDH) | F: TGGTGCTGACTATGTCGTGGA | — | — | — |
| | | R: CATGGGTGCATCTTTGCTTG | — | — | — |
| CA289286 | Aldehyde decarbonylase | F: GACCTTGTTCACCTCACGCA | D7 | 15.27 | 16.85 |
| | | R: ACGGTCTGGACGCTATGGA | D9 | 7.53 | 6.98 |
| CA110802 | Cinnamyl-alcohol dehydrogenase | F: AAGCACATTGGCGTAGTTGG | D7 | 36.96 | 34.31 |
| | | R: GACGGTCACCCTCATCCC | D9 | 40.26 | 38.26 |
| BU103703 | Delta-1-pyrroline-5-carboxylate synthetase | F: CCTAAAGCCAGGAAAGATAACA | D7 | 28.97 | 29.38 |
| | | R: AATAATGAGCAGAACACCCAAT | D9 | 27.91 | 26.89 |
| CA280103 | Serine/threonine-proteinkinase SRK2 | F: ACAGCAGGGCGATTCAGTG | D7 | 4.09 | 5.16 |
| | | R: TTCACGCGAGGTGTTGGA | D9 | 2.44 | 2.67 |
| DQ494704 | Phytochrome B | F: ATGGCAGTCATCATTAGCAGTG | D7 | 0.34 | 0.26 |
| | | R: GAAACTCGCAAGCATACCTCA | D9 | 0.41 | 0.35 |
| CA191067 | Two-component response regulator ARR-A family | F: ATGACGGTGGTGGATGCC | D7 | 0.13 | 0.24 |
| | | R: TCACTTGGTAGGACGAGTTCTTG | D9 | 0.15 | 0.27 |
| CA078060 | Protein phosphatase 2C | F: CGCGGACGAGTACAAGAAG | D3 | 16.30 | 15.36 |
| | | R: CGCGGCTTCATCATCCA | D7 | 14.23 | 15.26 |
| | | | D9 | 10.55 | 16.28 |
| CA196779 | Sucrose synthase | F: TGGAGGACCAGCTGAGAATA | D3 | 2.41 | 3.25 |
| | | R: CTCGAAGAAGTCAGCCATCAA | D7 | 3.85 | 4.06 |
| | | | D9 | 4.11 | 4.36 |
| CA134312 | Beta-glucosidase | F: CAAAGATTGTGGAGGCGTTTG | D3 | 3.96 | 3.15 |
| | | R: CCTCGGTTCGTTGAAGGTAAA | D7 | 2.78 | 2.68 |
| | | | D9 | 5.85 | 5.67 |
| CA258249 | Starch synthase | F: TACATTACATGGAAGGCAGACC | D3 | 0.48 | 0.37 |
| | | R: CTTTGAGACAAACCAGCCAAAG | D7 | 0.43 | 0.46 |
| | | | D9 | 0.47 | 0.39 |
| CA134928 | Rribose 5-phosphate isomerase A | F: GGCTAGACCACTACCTCCTAATC | D3 | 0.48 | 0.36 |
| | | R: CCAGCATCGGCCAAGTTTAT | | | |
| CA134623 | Asparagine synthase | F: CATCATCCCATCGTTTCCCTA | D7 | 10.27 | 9.85 |
| | | R: GTCTGTCATCAGCCGTTTTACC | | | |
| CA228575 | Tyrosine aminotransferase | F:CGGCTTGGATGGATAGTTACC | D9 | 2.55 | 2.63 |
| | | R: CTGGACAAATGTTGGAGGATC | | | |
| CA282232 | Phosphoribulokinase | F: GGAAAGGTGCTGCGTGTTA | D9 | 0.33 | 0.28 |
| | | R: TTTGATGCCAGGGTAAGAGC | | | |
| BU103680 | Inorganic pyrophosphatase | F: TGGAGTCCCTCGTGTTTGC | D9 | 3.21 | 3.11 |
| | | R: GGCGATTTGGCAGGAGTTA | | | |

Note: Primers of F/ R represent forward/ reverse primers; Time point indicated the sampling days; Number in microarray/qRT-PCR represented fold change of differential expressed genes in two testing methods, respectively.
